# Natural Molecules, Nutraceuticals, and Engineered Nanosystems: A Comprehensive Strategy for Combating *Gardnerella vaginalis*-Induced Bacterial Vaginosis

**DOI:** 10.3390/microorganisms13102411

**Published:** 2025-10-21

**Authors:** Manoj Dalabehera, Abdulrahman Mohammed Alhudhaibi, Emad M. Abdallah, Tarek H. Taha, Shubham Chaudhari, Alka Kumari, Rudra Narayan Subudhi, Charul Rathore

**Affiliations:** 1 Department of Pharmaceutics, Uttaranchal Institute of Pharmaceutical Sciences, Uttaranchal University, Dehradun 248007, Uttarakhand, India; manojdalabehera@uumail.in; 2 Department of Biology, College of Science, Imam Mohammad Ibn Saud Islamic University (IMSIU), Riyadh 11623, Saudi Arabia; thali@imamu.edu.sa; 3 Department of Biology, College of Science, Qassim University, Buraydah 51452, Saudi Arabia; 4 Institute of Pharma Sciences, J.S. University, Shikohabad 283135, Uttar Pradesh, India; sc902633@gmail.com; 5 University Institute of Pharma Sciences, Chandigarh University, Ajitgarh 140413, Punjab, India; alkakumari1221@gmail.com (A.K.); charulsingh29@yahoo.com (C.R.); 6 JBIT College of Pharmacy, 23 Milestone, NH-07, Chakrata Road, Shankarpur, Dehradun 248197, Uttarakhand, India; sipunsubudhi83@gmail.com

**Keywords:** antibacterial activity, bacterial vaginosis, plants, essential oils, probiotics, vitamins, proteins, fatty acids, enzymes, nanoformulation, *Gardnerella vaginalis*

## Abstract

Bacterial vaginosis (BV) is a highly prevalent vaginal infection characterized by a dysbiotic shift in the vaginal microbiota, with *Gardnerella vaginalis* acting as a principal pathogen. Despite its association with adverse reproductive outcomes, BV remains underexplored from both mechanistic and therapeutic standpoints. Standard antibiotic regimens frequently fail due to high recurrence rates driven by multidrug-resistant (MDR) *G. vaginalis* strains and biofilm formation. In response, natural compounds and nutraceuticals, owing to their intrinsic antibacterial, antibiofilm, and immunomodulatory properties, have emerged as promising candidates for alternative BV therapies. In this paper, we first compile and critically evaluate preclinical and clinical evidence on the efficacy of plant extracts, essential oils (EOs), probiotics, vitamins, proteins, fatty acids, and enzymes against *G. vaginalis*, emphasizing their mechanistic insights in restoring vaginal microbial balance. Next, we focus on the integration of these bioactive agents into engineered nanosystems, such as lipid-based nanoparticles (LNPs), polymeric carriers, and inorganic nanostructures, to overcome limitations related to solubility, stability, and targeted delivery. Nonetheless, comparative studies, combination therapies, and recent patent developments are discussed to highlight how naturally derived molecules can enhance antimicrobial potency and reduce cytotoxicity. In conclusion, these platforms demonstrate superior in vitro and in vivo efficacy, offering a paradigm shift in the management of BV. Key challenges include scalable manufacturing, regulatory approval, and comprehensive safety assessment. Future research should prioritize standardized nanoparticle (NP) synthesis, detailed pharmacokinetic and toxicity profiling, and well-designed clinical trials to validate nature-inspired, nanoengineered therapies against *G. vaginalis*-induced BV.

## 1. Introduction

As one of the most prevalent vaginal disorders worldwide, bacterial vaginosis (BV) affects nearly 30% of women of reproductive age and exhibits recurrence rates exceeding 50% within six months due to the rise in the antibiotic-resistant *Gardnerella vaginalis* strain [1,2]. Vaginal dysbiosis has been linked to an increased risk of HIV and other sexually transmitted infections, as well as pelvic inflammatory disease. Additionally, it has been connected to negative pregnancy outcomes, including preterm delivery and infections in both mothers and newborns [3].

BV is a dysbiosis of the vaginal microbiota that is defined by a change in the dominance of different anaerobic bacteria from *Lactobacilli* [4,5]. It is the most prevalent vaginal condition in women who are of reproductive ageworldwide and is identified as a clinical condition in which there is an overgrowth of bacteria in the vagina, resulting in a thin, gray/off-white, homogenous, and malodorous adherent secretion from the vagina [6,7,8]. This discharge is more noticeable after sexual activity and menstruation and has a pHhigher than 4.5 [9]. It also has an unpleasant fishy smell, with the patient’s most common complaint being itching in the perineal area [10,11]. Bacteria, namely *G. vaginalis*, *Atopobium vaginae*, *Megasphaera phylotypes*, *Leptotrichia aminionii*, *Mobiluncus* spp., *Prevotella* spp., *Mycoplasma hominis*, *Bacteroides* spp., and *Sneathia*, are among the causative spp. that have been found in recent studies to be responsible for BV [12,13].

*G. vaginalis* is also associated with adhering to vaginal epithelial cells, forming “clue cells,” which are diagnostic for BV. BV is often asymptomatic, but about half of affected individuals report malodor and mild irritation. This microorganism is a small, non-spore-forming, nonmotile *Coccobacillus* that displays Gram-variable staining due to its thin cell wall. *G. vaginalis* is known for biofilm formation, which enhances its persistence and resistance within the vaginal ecosystem. The bacterium produces virulence factors such as vaginolysin, proteases, and sialidase (SLD), contributing to tissue colonization and immune evasion.

Metronidazole (MTZ) is a first-line antibiotic for BV, but *G. vaginalis* exhibits considerable resistance, especially in recurrent cases. Approximately 68% of isolates resist MTZ, while most remain sensitive to clindamycin. Genomic studies have identified specific clades intrinsically resistant to MTZ, which correlates with treatment failures. Biofilm formation further contributes to poor antibiotic penetration and increases recurrence. Recurrence of BV, predominantly driven by *Gardnerella vaginalis*, is common following standard MTZ therapy. More than 50% of treated women experience recurrence within 6–12 months, particularly those with behavioral risk factors such as inconsistent condom use or retention of pretreatment sexual partners. Biofilm resilience and the presence of MTZ-resistant clades are significant contributors to recurrence.

It is projected that the global cost of treating symptomatic BV may reach $4.8 billion in the future, as it is the most frequent vaginal infection occurring in females [14,15,16]. Oral or intravaginal administration of the currently recommended first-line antimicrobial agents, including MTZ and clindamycin, is a widely used therapy for BV. These agents provide broad-spectrum activity against anaerobic bacteria associated with the condition [17]. However, due to limitations such as multidrug resistance (MDR), severe adverse events, and recurrence complaints, there is a demand for steering towards constituents derived from natural sources, which might stand a chance as an alternative approach [18,19,20]. Moreover, the integration of nanomaterials provides the desired therapeutic activity by sidestepping negative attributes associated with naturally based molecules, which may prove to be a useful asset in addressing the problem of BV recurrence [21,22].

The current study aims to outline and critically discuss emerging MDR patterns in *G. vaginalis*-induced BV, identify and characterize novel bioactive molecules from natural sources, and evaluate the potential of nano-enabled herbal formulations for targeted, sustained vaginal delivery. Additionally, we critically appraise clinical trial evidence on herbal and nutraceutical interventions to determine their efficacy, safety, and impact on recurrence rates, while mapping the global patent landscape to underscore recent innovations in this field.

## 2. Methodology

**Figure 1:**
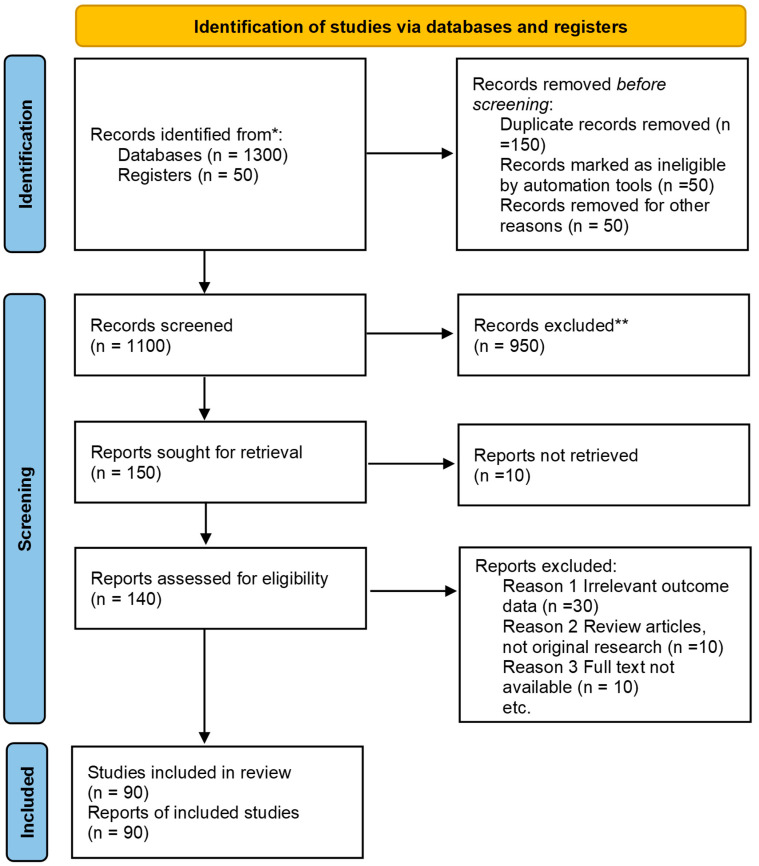
PRISMA flow diagram showing the identification, screening, eligibility assessment, and inclusion of studies in the systematic review.* Records Identified From, ** Records Excluded. (Registration No.CRD420251089821, PRISMA checklist in the Table S1). This work is licensed under CC BY 4.0. https://creativecommons.org/licenses/by/4.0/.

In this paper, we performed a systematic literature review (Registration No.CRD420251089821, PRISMA checklist in the Table S1) of peer-reviewed articles, clinical trial reports, and patent filings published up to May 2025, sourcing records from The Lancet, PubMed, Springer, ScienceDirect, Nature, Taylor and Francis, Google Scholar, the WIPO PatentScope database, and major clinical trial registries. Search terms included “bacterial vaginosis,” “*G. vaginalis*,” “antibacterial resistance,” “natural bioactives,” “nutraceuticals,” “nanoherbal formulations,” “herbal clinical trials,” and “patents.” We included original research articles, in vitro and in vivo studies, human clinical trials, and patent documents that explicitly investigated antimicrobial activity against *G. vaginalis* or BV recurrence, design, and evaluation of nano-enabled herbal delivery systems (Figure 1).

Exclusion criteria comprised non-English publications, conference abstracts lacking full text access, studies focused on non-vaginal pathogens or synthetic compounds only, articles with low methodological quality, and patents without demonstrable utility data. Retrieved records were screened by title and abstract, and full texts were assessed independently by two reviewers; disagreements were resolved through consensus.

## 3. Mechanisms of *Gardnerella vaginalis* Pathogenicity

**Figure 2:**
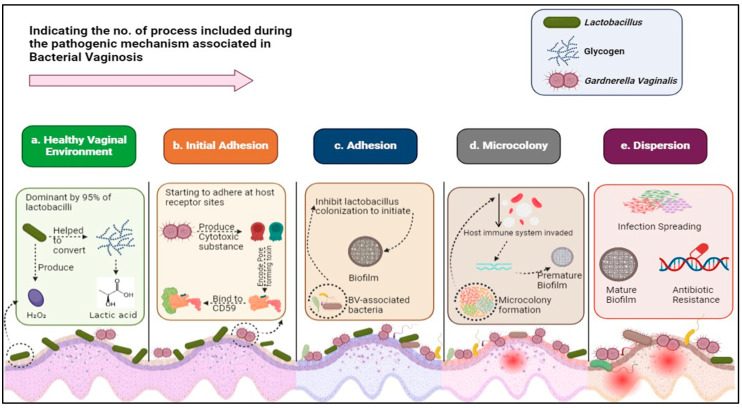
Represents a schematic illustration of the BV pathogenic mechanism. (**a**)—Step 1: *Lactobacillus* dominates other aerobic and anaerobic bacteria in a healthy vagina. (**b**)—Step 2: Initial adhesion of pathogenic bacteria like *G. vaginalis* and *P. bivia* occurs, suppressing the host immune system. (**c**)—Step 3: BVAB starts to inhibit Lactobacillus colonization to initiate biofilm. (**d**)—Step 4:Pathogenic bacteria form a microcolony in the vagina by converting its medium from acidic to alkaline. (**e**)—Step 5: Mature biofilm forms, and infection spreads to the nearby epithelium in the vagina. Generated by Biorender (2025) https://app.biorender.com/, [Date accessed: 10 March 2025].

BV is also known as nonspecific *G. vaginalis*, and its pathogenic mechanism occurs due to the replacement or displacement of *Lactobacillus* spp. by an increase of 100 to 1000 times of various aerobic and anaerobic facultative bacteria; however, *G. vaginalis* is primarily involved throughout the process [23,24]. In a healthy vagina, *Lactobacillus* maintains the homeostasis of vaginal microbiota by converting glycogen (produced by vaginal epithelial cells with the assistance of α amylase) into lactic acid (LA), which further helps maintain an acidic medium in the vagina [4,25]. An acidic medium is highly suitable for preventing bacterial overgrowth; moreover, *Lactobacillus* spp. also produces hydrogen peroxide (H_2_O_2_), which inhibits other anaerobic bacteria, including *G. vaginalis*, as it is an important element in the vaginal defense system [26,27]. *Lactobacillus* also enhances vaginal flora by producing antimicrobial and anti-inflammatory products like bacteriocins and biosurfactants (Figure 2, step 1) [28].

Research indicates that several risk factors and behaviors are linked to pathogenic initiation of BV, such as age, marital and employment status, occupation, multiple exposure of antibiotics, reduced host estrogen production, sexual activity, younger age at first intercourse, more frequent episodes to receptive oral sex, use of spermicides, STDs, working in the sex industry, contraceptive use, frequency of vaginal intercourse, and race/ethnicity [1,29,30,31,32]. In step 2, *G. vaginalis* and other BV-associated bacteria (BVAB), like *Prevotella*, *Mycoplasma*, *Ureaplasma*, and *Mobiluncus* spp., initially adhere to the vaginal epithelium and produce vaginolysin and cholesterol-dependent cytolysin, which helps suppress the host’s innate immune response [33,34]. These cytotoxic substances further encode a pore-forming toxin, which binds the host’s complementary regulatory molecule CD59 [35,36]. In step 3, *G. vaginalis* promotes *P. bivia* growth, and these two bacteria produce an enzyme like SLD, which degrades the vaginal epithelium mucin layer [37]. Bacterial adhesion interacts between cell appendages, carbohydrates, and cell surfaces to initiate biofilm while inhibiting *Lactobacillus* colonization in the vaginal micro-ecosystem [38,39]. During this process, theBapL protein in *G. vaginalis* is highly expressed, which helps to initiate bacterial biofilm production [28,40]. In step 4, in the early stage, *G. vaginalis* and *Prevotella* are first to initiate microcolony formation while continuously changing normal *Lactobacillus*, which later helps to facilitate BVAB to provide a simultaneously anaerobic vaginal environment to favorably disrupt the host epithelial barrier [41]. After the successful formation of a microcolony, extracellular DNA in *G. vaginalis* stimulates the production of extracellular matrix (EPS), which further provides structural stability and integrity to biofilm [42,43]. This colony formation jeopardized the host’s inflammatory and immune system to the biofilms [41]. In step 5, infections are ready to spread [44]. Biofilm helps bacteria by not allowing them to be fully killed or removed by antibiotics and the host immune system; therefore, the persistent presence of bacterial biofilm further causes the recurrence of BV [45,46]. In this stage, clinical symptoms of BV, including vaginal discharge, appear in women’s vaginas (Figure 2) [25,40,47].

## 4. Characterizing the Resistance Profile of *Gardnerella vaginalis*

**Figure 3:**
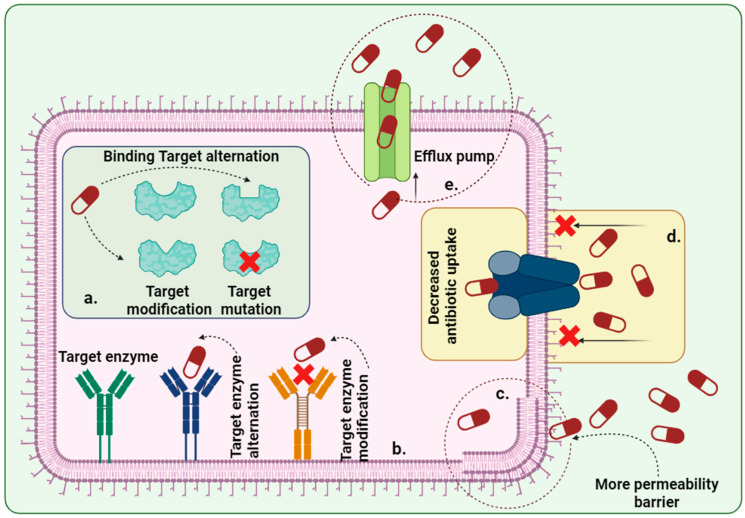
A schematic illustration of current standard antibiotics developing resistance in BV. (**a**)—Antibiotic binding sites were altered, and mutations occurred in the bacterial cell membrane. (**b**)—Target enzyme modified and altered. (**c**)—Due to the bacterial membrane’s more permeable nature, antibiotics hardly enter and show their effect. (**d**)—Decreased antibiotic uptake. (**e**)—Drug constituents are pumped out to the outer site through the efflux pump. Biorender (2025) https://app.biorender.com/, [Date accessed: 11 March 2025].

Various currently available marketed medicines are used for the management of BV. However, due to multiple exposures and fast-developing resistance, it is necessary to adapt natural-based constituents and integrate them with nanomaterials for better therapeutic purposes [9,39]. Table 1 lists a compilation of current drug formulations with various adverse events. According to various established reports, bacterial resistance occurred due to drug molecules not reaching target sites and target enzymes because of target site, enzyme modification, and mutation, respectively [48]. Another reason for resistance is the pumping out of antibiotic constituents from the bacterial cellular membrane through efflux pumps and decreasing their uptake, so that the concentrations of drug molecules decrease and their antibacterial efficacy [49,50]. Bacterial membrane permeability is also an important factor for drug resistance, leading to a smaller amount of drug entering the membrane (Figure 3) [51].

## 5. Naturally Derived Agents Against BV

There are various molecules derived from natural sources, such as plants and microorganisms, that have been proven to have promising therapeutic activity in BV (Table 1). Moreover, Table 2 lists the synergistic activity of various naturally sourced molecules with potential relevance to *G. vaginalis*.

### 5.1. Synergistic Applications of Herbal Therapies

#### 5.1.1. Live Bacteria

Naturally sourced constituents and their combination have emerged as having promising antibacterial activity, which outcomes could sidestep current conventional therapies’ associated side effects and MDR [63,64,65]. However, synergistic data, specifically if steering into molecules derived from natural sources, has not yet been explored across BV. In this section, we have compiled various studies. For instance, Choi et al. [64] investigated the effect of *Lactobacilli* strains (*Ligilactobacillus salivarius*, *Limosilactobacillus fermentum*, *Lactiplantibacillus plantarum*, *Lacticaseibacillus paracasei*, and *Lacticaseibacillus rhamnosus*) in HeLa cells and a *G. vaginalis*-infected mouse model. In this study, it has been demonstrated that maximum production of H_2_O_2_ and LA was observed with inhibition of *G. vaginalis* growth up to 80%. This combination was orally administered to infected C57BL/6 mice at 5 × 10^8^ and 5 × 10^9^ colony-forming units (CFUs)/mouse daily for 2 weeks. The higher dose notably alleviated *G. vaginalis* counts in the vaginal tract, reduced neutrophil-associated myeloperoxidase (MPO) activity, and suppressed pro-inflammatory cytokines. Histopathological analysis confirmed the inhibition of vaginal epithelial cell exfoliation without any side effects occurring, which highlights its potential insights in BV [64]. Sabbatini et al., 2020, first demonstrated the antibiofilm properties of *Saccharomyces cerevisiae* and *L. rhamnosus* combination. In their study, *G. vaginalis* biofilm cell inhibition and disaggregation of *S. cerevisiae* at 10^7^–10^8^ CFU/mL of prepared biofilm were observed after combination therapy administration. Moreover, *S. cerevisiae* (10^6^ CFU/mL) with MTZ (4 µg/mL) enhanced antibiotic activity. This therapy outlines its mechanism by proteases and inhibition of SLD [65]. Similarly, Jang et al., 2017 [66] investigated oral administration of a probiotic mixture (PM) containing HN001 (L1), Gla 14 (L2), and *lactoferrin* RCXTM either orally or intravaginally over 14 days at doses of 5 × 10^8^ to 5 × 10^9^ CFU/mouse/day. This investigation aims to evaluate the significant suppression of vaginal dysbiosis. Oral and intravaginal administration of probiotic mixtures has led to the potential alleviation of *G. vaginalis*-induced cell disruption in vaginal epithelial cells, demonstrating its anti-BV efficacy without any side effects being noticed. Moreover, compared to vaginal administration, oral administration of PM was found to be more effective in terms of regulating both vaginal and systemic innate modulating (suppression of NF κB activation, MPO activity) and adaptive immunity (downregulation of Th17 marker RORγt and upregulation of Treg marker Foxp3) [66]. This clinical study demonstrated a combination of probiotics with conventional treatment for BV and aerobic vaginitis. Heczko et al., 2015, evaluated 578 women with recurrent episodes of BVAV. Participants were orally administered (500 mg twice daily for 7 days) plus either a probiotic (prOVag^®^) or a placebo. The probiotic contained three strains: *L. fermentum 57A*, *L. plantarum 57B*, and *L. gasseri 57C* (≥10^8^ CFU). Results showed that probiotic supplementation extended the symptom-free interval by up to 51% (mean 71.4 vs. 47.3 days, *p* = 0.0125), with potential actions (76%) in patients with MTZ-resistant *G. vaginalis* (*p* = 0.0053). Additionally, the probiotic group demonstrated speedy recovery of *Lactobacillus* counts, compared to the placebo [67].

#### 5.1.2. Natural Molecules

Scuderi et al., 2023, have investigated the therapeutic efficacy of pea protein, grape seedextract, and LA combination (daily intravaginal dose of 0.2 g/mouse for 7 days) and its mechanistic insights. The results revealed a significant reduction in *G. vaginalis* proliferation and a significant reduction (35.85% in CFU for pretreatment vs. 11% for posttreatment) in the number of neutrophil infiltrations, SLD activity, and inflammatory markers. Due to its bioadhesive property, the product containing grape seed extract, LA, and pea protein can be used as an alternative treatment approach in the future [68]. Donkor et al., 2023, have demonstrated the combined activity of *Senna alata*, *Ricinus communis*, and *Lannea barteri* extracts, and the minimum inhibitory concentration (MIC) of this was found to be significantly lowered as compared to the individual value. Notably, combinations like *L. barteri aqueous + S. alata ethanol* and *S. alata aqueous + R. communis ethanol* showed synergy (FICI ≤ 0.5). For *E. coli* (0.97 to 1.17 mg/mL), *S. aureus* (0.97 to 4.69 mg/mL), *P. aeruginosa* (0.50 to 1.17 mg/mL), *K. pneumonia* (1.17 to 3.12 mg/mL) and *C. albicans* (2.34 to 4.69 mg/mL), it was observed that they might stand a chance as promising antibacterial therapy without any antagonistic interactions [69]. Sousa et al. [70] demonstrated the antibacterial efficacy of *Thymbra capitata* essential oils (EOs). To study the potential synergistic effect, two EOs of *T. capitata* were characterized to test their antimicrobial activities. It was found that carvacrol (MIC ~0.04–0.08 µL/mL) and ρ-cymene exhibited a potent synergistic antibacterial effect towards *Gardnerella* spp. Carvacrol and linalool at sub-MICs were revealed to be more efficient in the elimination of biofilm cells, but no cytotoxicity was found, further extending exploration necessity [70]. Strydom et al. [71] designed a randomized, double-blind, placebo-controlled feasibility trial on intravaginal combination therapy of 3% acetic acid and 2% LA, and the active groups were noted. A notable reduction in symptomatic recurrence, from a baseline annual mean of 7.9 episodes to ~1 episode, andgood tolerance was noticed during this intervention. Nonetheless, compared to conventional fluconazole (150 mg weekly), which often demonstrates > 50% relapse (denotes the return of infection due to incomplete eradication of the initial causative organism) within 3–6 months and involves systemic side effects, this combination therapy offers a localized, low-risk, drug-free alternative [71]. Noll et al., 2012, in their study, first investigated the combination effect against *G. vaginalis*, which is a causative agent for BV. It was investigated that subtilosin had a synergistic effect with antimicrobial agents, and it was effective in inhibiting the growth of GV [72]. Mazhar et al., 2017, investigated the safety and therapeutic efficacy of a cranberry extract, *Bacillus coagulans*, and turmeric extract combination to prevent recurrent episodes. Results showed that the recurrence (referring to new episodes of infection occurring after successful treatment) rate was significantly reduced after administration of combination therapy for 12 weeks [73]. Motlagh A et al., 2017, investigated the efficacy of *Prangos ferulacea*-based vaginal cream on patients suffering from BV. Authors have demonstrated the elevation of the recovery rate after administration and suppressed instances of resistance to *G. vaginalis*, which highlights its potential and could be used as an alternative to current medications like MTZ [74]. Algburiet al., 2015, investigated the synergistic effect of subtilosin and lauramide arginine ethyl ester (LAE) against biofilms of *G. vaginalis*. When used alone, the minimum bactericidal concentration (MBC B) of clindamycin towards *G. vaginalis* was 20,000 μg/mL, and MTZ was 500 μg/mL. In contrast, combining clindamycin with subtilosin reduced its MBC B more than sixfold (to 2.9 mg/mL), and MTZ combined with subtilosin showed an eightfold reduction in required dose (62.5 μg/mL vs. 500 μg/mL). Similarly, LAE suppressed the doses of antibiotics by enhancing permeability and biofilm penetration. Notably, these combinations inhibit healthy vaginal *Lactobacilli* biofilms, a significant advantage over antibiotics alone [75]. Noll et al., 2012, first demonstrated the anti-*G*. *vaginalis* activity of subtilosin, LAE, e-poly-L-lysine, clindamycin phosphate, and the MTZ combination, and in their study, they observed that significant growth inhibition was noticed with a lower risk of bacterial resistance at lower MIC (2–25 μg/mL). The triple combinations (e.g., subtilosin + LAE + 0.5 × MIC GML) maintained synergy while preserving *Lactobacillus* viability and significantly inhibiting other pathogens. Mechanistically, these compounds employ membrane disruption, signal interference, and pore formation [76].

**Table 2:** List of herbal combination data concerning *G. vaginalis*.

Sl. No.	Combination	Performed Assay/Method	Strains Used	Types of Study	Outcomes	References
Live Bacteria						
1.	*Ligilactobacillus salivarius* MG242, *Limosilactobacillus fermentum* MG901, *Lactiplantibacillus plantarum* MG989, *Lacticaseibacillus paracasei* MG4272, and *Lacticaseibacillus rhamnosus* MG4288	Detection of H_2_O_2_ Production Study, Analysis of LA Production, Antibacterial Effects of *Lactobacilli* Strains on *G. vaginalis* Growth, e Cytotoxic Effect Assay, Adhesion Ability of *Lactobacilli* Strains to HeLa, MPO Activity in Vaginal Lysates	*G. vaginalis* (KCTC5096)	In vitro (HeLa cell line used) and In Vivo (C57BL/6)	Synergistic potential against *G. vaginalis* and significant inhibition of vaginal epithelial exfoliation were observed.	[64]
2.	*S. cerevisiae* CNCM I-3856 and *Lacticaseibacillus rhamnosus* ATCC 53103 probiotic	Inhibition and Disaggregation of *G. vaginalis* Biofilm Study, Co-Aggregation Assay, Anti-Biofilm Activity of MTZ and Clindamycin	*Streptomycin*-resistant *G. vaginalis*	In vitro	Promising inhibition and disruption of bacterial biofilm were observed.	[65]
3.	*L. rhamnosus* HN001 and *L. acidophilus* GLa-14	Assay for the Inhibitory Effects of Probiotics Against the Growth of *G. vaginalis* and AV, Assay for the Antagonistic Effects of Probiotics on the Adherence of *G. vaginalis* to HeLa Cells, MPO Activity Assay, ELISA	*G. vaginalis* KCTC5096 and *Atopobium vaginae* KCTC15240	In vitro (HeLa cells) and In vivo (C57BL/6)	Probiotics comprising a combination of this spp. exhibited desired killing activity against *G. vaginalis* with anti-inflammatory characteristics.	[66]
4.	MTZ and oral probiotics (prOVag)	Antibacterial Study	*G. vaginalis*	Multicentre, randomized, double-blind, placebo controlled trial (NCT01993524)	Clinical and microbiological parameters of BV were improved by this combination.	[67]
Natural Molecules						
1.	*Pea protein*, *Grape seed* extract, and LA	CFU Evaluation, MPO Activity, SLD Activity Assay, MucoadhesionStudy	*G. vaginalis* (KCTC5096)	In vivo (C57BL/6 mice model)	This combination improved vaginal tissue architecture while suppressing bacterial invasion.	[68]
2.	*Senna alata*, *Ricinus communis*, and *Lannea barteri*	Antimicrobial Bioassay, Synergy Analysis	*E. coli*	In vitro	Additive antibacterial activity was demonstrated.	[69]
3.	Carvacrol, α-terpinene, γ-terpinene, ρ-cymene, and linalool	Vaginal Irritation Test, Biofilm Biomass Quantification by Crystal Violet Staining Method, Checkerboard Method for Fractional Inhibitory Concentration	*G. vaginalis* UM137, *G. piotii* UM035, *G. leopoldii* UGent 09.48, and *G. swidsinskii* GS 9838-1	In vitro (Human Vaginal Epithelium (HVESkinEthic)	No cytotoxicity was observed in the tested vaginal tissue.	[70]
4.	Acetic acid and LA (intravaginal combination therapy)	Antimicrobial Study	Patients with vaginal infection	Double blinded randomized controlled feasibility trial (ACTRN 12620001084976)	The BV recurrence rate had drastically reduced.	[71]
5.	Subtilosin and glycerol monolaurate	Checkerboard Assays, Antimicrobial Assay	*Gardnerella vaginalis* ATCC 14018, *Peptostreptococcus anaerobius* ATCC 27337, and *Mobiluncus curtisii* ATCC 35241	In vitro (first time performed)	From MIC 4.6–25 μg/mL, this herbal combination showed 4-fold growth inhibition of *G. vaginalis*.	[72]
6.	*Cranberry* extract, *Bacillus coagulans*, and *Turmeric* extract	Antibacterial Study, Recurrence Assay	Patients with BV	Observational/descriptive study.	76.9% of patients improved after exposure to combination therapy.	[73]
7.	*Prangos ferulacea* vaginal cream with MTZ	Antibacterial Study	Patients suffered from BV	Randomized controlled clinical trial (IRCT2016042327534N1)	Recovery of BV in patients was observed.	[74]
8.	Subtilosin and LAE	Bacterial Biofilm Formation Assay, Time Bactericidal Activity of Antimicrobials Against Biofilm-Associated *G. vaginalis*, Plate Counting Method, Checkerboard Assay	*G. vaginalis* ATCC 14018	In vitro	Effective inhibition of *G. vaginalis* biofilm observed without affecting vaginal *Lactobacilli* at minimum bactericidal concentrations for biofilm cells (MBCs B) of 50 µg mL^−1^ (LAE), 69.5 µg mL^−1^ (subtilosin).	[75]
9.	LAE, e-poly-L-lysine, clindamycin phosphate, and MTZ	Determination of Minimal Inhibitory Concentration (MICs), Checkerboard Assays, and Antibacterial Study	*Gardnerella vaginalis* ATCC 14018, clinical isolates in healthy women (*Lactobacillus gasseri* ATCC 33323 and *L. plantarum* ATCC 39268), and those with recurrent (*L. acidophilus* ATCC 4356 and *L. vaginalis* ATCC 49540)	In vitro	Potential antibacterial activity was noticed.	[76]

MTZ—Metronidazole, MPO—Myeloperoxidase, LA—Lactic Acid, CFU—Colony-Forming Unit, SLD—Sialidase, BV—Bacterial Vaginosis, MBC—Minimum Bactericidal Concentration.

### 5.2. Comparative Study of Natural vs. Synthetic Drugs for BV

Comparative data play a crucial role in understanding potent anti-*G*. *vaginalis* action between constituents derived from natural sources and conventional therapies. Moreover, this paragraph of the paper critically analyzed the comparative insights and the necessity of using natural molecules (Table 3).

Khazaeian et al. [77] designed a triple-blind, parallel, randomized clinical trial by randomly allocating subjects into two groups, such as *n* = 35 (sucrose vaginal gel) and *n* = 35 (MTZ vaginal gel), to compare their antibacterial effect. Outcomes of their study demonstrated that the presence of vaginal discharge and clue cells was reported to be comparatively suppressed after administration of sucrose gel compared to antibiotic gel comprising MTZ. It was also noticed that both vaginal gels have significantly similar potential; however, the sucrose vaginal gel showed no side effects compared to the conventional one, which significantly highlights its significance as an alternative therapy [77]. Baig et al. [78] compared the anti-*G. vaginalis* efficacy of Kakrasingi (*n* = 31) as the intervention group and MTZ (*n* = 31) as the standard group patients by conducting a randomized (1:1), standard-controlled, single-center study. In their study, investigators noticed that clinical (54.83%), microbiological (51.61%), and therapeutic (51.61%) cure rates improved in the intervention group of patients compared to MTZ (51.61%, 45.16%, and 45.16%), respectively. Although both Kakrasingiand MTZ showed good efficacy, the herbal powder showed effects with no adverse effects, which depicts its promising efficacy compared to conventional [78].

A double-blind, randomized controlled trial was conducted by Afzali et al., 2020, to assess the anti-*G. vaginalis* potential of *Quercus brantii Lindl*-loaded vaginal cream (*n* = 42) and MTZ vaginal gel (*n* = 42) on reproductive-aged women. Findings of their study were that Quercus vaginal cream showed comparatively more effective potential than gel comprising MTZ, without any side effects. Investigators have also concluded that, for reproductive-aged women, oak gall herbs could give desired antibacterial action and are safely recommended, as three patients who administered MTZ gel exhibited effects like vaginal burning [79]. Similarly, Zare et al., 2018, compared, for the first time, the effects of vaginal cream containing *Quercus brantii Lindl*. (*n* = 84) with the MTZ tablet (*n* = 84) on BV by conducting a randomized clinical trial. Within their investigation, it was found that 73.8% of patients benefited (relieved from clinical symptoms like vaginal discharge, itching, erythema, edema, etc.) after exposure to the herbal cream without any side effects, whereas in the MTZ group, there were only 50% of patients who benefited after the treatment. They have also concluded that *Q. Lindl* is a potential herb that could be further studied and alternatively used in the future for desired antibacterial effects [80].

Durićet al., 2021, assessed a controlled clinical study by randomly allocating women into two groups (Group A—113 and Group B—97) by preparing formulations like VagitoryA (*Calendulaeextractumoleosum*, *Bursae pastoris*, *Hypericiextractum*, *Millefoliiextractum*), VagitoryB (TTO), and VagitoryC (*H. extractum*). In this present study, researchers have compared these herbal vagitories with each other and probiotics to characterize their antibacterial efficacy in BV. From the results data, it was found that only mild dryness in the vagina was observed in women after contact with TTO administration. Except for that, all vagitories exhibited positive effects on clinical symptoms like vaginal discharge, itching, redness, etc., with no reported serious side effects [81]. Masoudi et al., 2016, have compared the antibacterial therapeutic potential of *Myrtus communis* and *Berberis vulgaris* with MTZ vaginal gel in infected married women by designing a randomized clinical trial. Findings in their study exhibited that the group of patients in *M. communis* and *B. vulgaris* reported an effective response than MTZ. Authors also concluded that this herbal-based gel showed desired antibacterial action without any relapse and could be used further, while 30% of patients receiving MTZ experienced a relapse action [82]. Shabanianet al., 2019, designed a double-blind clinical trial to compare the antibacterial potential of *B. vulgaris* gel (intervention group) with MTZ gel by allocating women into two groups. Results in the present study showed that patients taking the herbal-based gel reported a continuous improvement of symptoms after 7 days than the synthetic drug. Moreover, after 21 days of intervention, total removal of symptoms associated with recurrent episodes was observed by the researcher. Investigators also demonstrated that *B. vulgaris* gel showed potential antibacterial effects and could be used in the future across vaginal infections [83]. The present study aims to compare the therapeutic antibacterial efficacy of garlic-loaded tablets with oral MTZ by designing a randomized controlled clinical trial on 120 married women. Outcomes from their study unfolded as both treatments showed better effects, but considering side effects and complications associated with MTZ, the investigator concluded that herbal-based therapy could be a potential alternative in the future (Mohammadzadeh et al., 2014) [84]. Wijgert et al., 2020, performed a comparative study between vaginal probiotics containing *Lactobacilli* with standard MTZ-based treatment to evaluate their potential in recurrence episodes. In their study, lower recurrence rates were observed after probiotic exposure, as it is known to improve vaginal microbiota [76]. Hakimi et al., 2017, conducted a triple-blind randomized controlled study to compare a probiotic-loaded vaginal gel with an oral MTZ tablet in recurrent cases. Results exhibited that, after 90 days of probiotic treatment, an 84% healing rate was observed in women, whereas only 62% improvement was noticed in the MTZ group [85]. Critically comparing this to conventional treatments, antibiotics like MTZ or clindamycin, though effective in short-term pathogen clearance, often lead to recurrence rates exceeding 50% due to disruption of normal flora and lack of microbiota restoration. In contrast, LM5 addresses both infection control and ecological restoration of the vaginal microbiome. These natural molecules support the superior mucosal protection, anti-inflammatory activity, and biofilm disruption capability of natural agents over conventional antibiotics. Importantly, these natural compounds did not damage reconstituted human vaginal epithelial tissues, and no bacterial recovery was observed even after 24 h in fresh medium, suggesting long-lasting antimicrobial action.

## 6. Nutraceutical Approaches to BV: Focus on Probiotic Strains (*L. gasseri*, *L. helveticus*, *L. rhamnosus*, *L. acidophilus*, *L. plantarum*, *L. delbrueckii*, *L. fermentum*, and *L. salivarius*)

According to various established reports, nutraceuticals nowadays have exhibited desired antibacterial efficacy in vaginal infections, i.e., BV [86,87,88]. Daily life nutraceuticals have emerged as having promising anti-*G*. *vaginalis* efficacy by maintaining vaginal microflora; therefore, in this section, we have discussed potential nutraceuticals and their mechanistic insights towards *G. vaginalis* induction. For instance, Zhang et al., 2022, have demonstrated in vitro and in vivo anti-*G. vaginalis* inhibitory activity of *L. gasseri* spp. From the in vitro assay, it was revealed that adhesion ability and biofilm formation on the vaginal epithelium were effectively inhibited by *L. gasseri*. *G. vaginalis* SLD activity suppression and modulation of interleukin-1β and TNF-α have been observed in an in vivo study. Investigators also concluded that *L. gasseri* could be a potential alternative treatment option, as it enhances vaginal tissue inflammatory cell infiltration (Table 4) [89].

Kim et al., 2022, have demonstrated in vitro and in vivo antibacterial mechanisms of *L. helveticus* (HY7801) towards *G. vaginalis* biofilm formation and epithelial cell adhesion.An in vivoassay confirmed that pro-inflammatory cytokines and *G. vaginalis* counts in vaginal mucosa have been efficiently suppressed in murine models after exposure to HY7801. This *Lactobacillus* spp. also helped to boost H_2_O_2_ and maximum LA production in the vagina, observed in an in vitro assay, and downregulated the expression of genes associated with *G. vaginalis* virulence properties, hence evident to be a promising candidate for BV [90]. Attasi et al., 2010, have evaluated the anti-*G. vaginalis* potential of LA and demonstrated that, at 100 mM concentration, *G. vaginalis* showedpromising killing activity. It was also observed by LA, at a 65 mM concentration; LA enhances significant *G. vaginalis* killing activity of H_2_O_2_ [91]. Li et al., 2023, have loaded LAB strains such as *L. crispatus*, *L. rhamnosus*, *L. salivarius*, and *L. plantarum* into bacterial consortia transplantation to overcome *G. vaginalis*-induced BV. This transplantation successfully produced adequate LA and H_2_O_2_, hence regulating the vaginal microbiota. The authors concluded that this might be a potential treatment across BV, but still, more studies are needed to carry out for its successful implementation [92].

Zhi Tan et al., 2024, have investigated the correlation between serum carotenoids like α-carotene, β-carotene, β-cryptoxanthin, lycopene, and lutein/zeaxanthin and the development of BV. Findings showed that all serum carotenoids exhibited an inverse association. It was also noticed that a comparatively lower incidence of BV was shown by α-carotene and β-cryptoxanthin, and β-carotene, followed by a negative correlation amongst all carotenoids [93]. Vivekanandan et al., 2024, have evaluated VagiBIOM *Lactobacillus* suppository by designing a randomized, double-blind, placebo-controlled pilot study involving patients. Results exhibited that clinical symptoms significantly returned to baseline after a 28-day intervention of herbal therapy administration. Authors also concluded that this VagiBIOM suppository holds tremendous antibacterial activity, which could be an alternative to current standard medication [94]. Sabbatini et al., 2018, have first demonstrated an in vitro and in vivo assay to evaluate the ability of *S. cerevisiae*-based probiotics against *G. vaginalis*-induced infections. From the murine model outcome study, it was noticed that SLD and epithelial exfoliation activity effectively inhibited and suppressed *G. vaginalis* adherence to vaginal mucosa and reduced inflammation without any side effects noticed. Hence, it could be a potential therapeutic alternative and could be used in the future for better outcomes [95]. Lin et al., 2021, have investigated the in vitro antibacterial efficacy of four *Lactobacillus* strains (*L. rhamnosus*, *L. acidophilus*, *L. rhamnosus*, and *L. plantarum*), each administered at 1 × 10^10^ CFU daily against *G. vaginalis.* These treatments exhibited statistically significant reductions in Nugent scores over 4 weeks (e.g., GMNL-74 and GMNL-185 mean reduction from 5.44 to below 3, *p* < 0.01), with improved clinical symptoms like vaginal discharge color, odor, and itching. From the antimicrobial assay, it was noticed that maximum inhibition (18.27 ± 3.02) and adherence of bacteria to HeLa were exhibited. Additionally, GMNL-74 and GMNL-185 strains comparatively inhibit maximum *G. vaginalis* adherence capacity and hold potential antibacterial activity against *G. vaginalis* and could be a suitable alternative therapy to conventional [96].

**Table 4:** Enlists the Probiotic Strains in the Treatment of BV.

Sl. No.	*Lactobacillus* Strains (Probiotics)		Types of Performed Study	Experimental Types	Antibacterial Against	Key Findings	References
1.	*L. gasseri* CCFM1201		1. AntiSLD assay 2. Antibiofilm study 3. Histopathological examination	In vitro (HeLa) and in vivo (murine model)	*G. vaginalis*	Potential improvement of Vaginal epithelial cell exfoliation observed.	[89]
2.	*L. helveticus* HY7801		1. Measurement of cytokines in vaginal tissues 2. Histopathological examination 3. Adhesion and biofilm assay 4. Antibacterial study 5. Determination of H_2_O_2_ and organic acid production 6. Effect of HY7801 on *G. vaginalis* virulence gene expression	In vitro (HeLaand in vivo (C57BL/6)	*G. vaginalis* ATCC14018	This probiotics strain provided the desired anti-*G. vaginalis* activity.	[90]
3.	Serum carotenoids		Antibacterial study	Cross-sectional	Patients (1252) suffered from BV	Serum carotenoids were found to be negatively associated with BV.	[93]
4.	VagiBIOM *Lactobacillus*		1. Vaginal swabs, DNA isolation, and microbiome analysis 2. Statistical and bioinformatics analysis	Randomized, double-blind, placebo-controlled pilot study (NCT05060029)	Patients (92)	Beneficial effects on the vaginal microbiome are demonstrated.	[94]
5.	*S. cerevisiae*		1. SLD activity assay 2. Adhesion and displacement assays 3. Co-aggregation assay	In vitro (A-431 and HeLa cell lines) andin vivo (C57/Bl6 female mice)	*G. vaginalis* clinical isolates	Significant inhibition of *G. vaginalis* was investigated.	[95]
7.	*L. rhamnosus*, *L. acidophilus*, *L. rhamnosus* and *L. plantarum*		1. Antibacterial activity of *Lactobacilli* 2. Adherence of bacteria to HeLa 3. Clinical study protocol and sample collection 4. RNA Extraction and quantitative reverse transcription polymerase chain reaction (qRT PCR) analysis of inflammatory cytokines 5. RNA extraction and qRT PCR analysis of inflammatory cytokines 6. DNA extraction and vaginal microbiota analysis	In vitro (HeLa cell line), a randomized, double-blinded trial (NCT 03116789)	*G. vaginalis* (BCRC 17040), *E. coli* (BCRC 11634)	Promising bacterial adhesion inhibition observed.	[96]
8.	*L. delbrueckii* DM8909, *L. plantarum* ATCC14917, and *L. plantarum* ZX27		1. Co-aggregation assay 2. Co-culture assay 3. Evaluation of *Lactobacillus* sp. for antagonism of *G. vaginalis* adhesion and biofilm formation and preformation 4. Impact of *Lactobacillus* sp. CFS on *G. vaginalis* gene expression 5. Anti-inflammatory Effects of Probiotic Bacteria on HeLa	In vitro (HeLa cell line)	*G. vaginalis*. ATCC49145	This study demonstrated that gene expression related to *G. vaginalis* biofilm has been potentially downregulated.	[97]
9.	*L. paracasei* CH88		1. Evaluation of the ameliorative effect of *L. paracasei* CH88 and the <3 kDa LCFSP on BV-induced mice 2. Evaluation of *G. vaginalis* CFU in vaginal fluid 3. Histopathological examination	In vivo (Female C57BL/6 mice)	*G. vaginalis* KCTC 5097	*G. vaginalis* biofilm properties were inhibited.	[98]
10.	Probiotic	*L. salivarius* MG242, *L. fermentum* MG901, and *L. plantarum* MG989	1. Histopathological analysis 2. Adhesion 3. Antibiotic susceptibility 4. Assessment of enzyme production 5. Analysis of the LA level using the HPLC UV method 6. Hemolysis activity 7. Bile salt hydrolase activity	In vivo (seven-week-old 57BL/6J female mice)	*G. vaginalis* KCTC5096	*G. vaginalis*-related vaginal colonies were significantly reduced.	[99]
		*L. fermentum* 5.2, *L. plantarum* 6.2, and *L. plantarum* 7.1	1. Auto-aggregation and co-aggregation assays 2. Microbial hydrophobicity assay 3. Lactobacillus adhesion to HMVII cells 4. Antimicrobial activity of *Lactobacillus* culture supernatants	In vitro (HMVII, a vaginal epithelial cell line (BCRJ 0316))	*G. vaginalis* ATCC 49154	Vaginal microbiome improved.	[100]
		*L. plantarum* strains (ZX1, ZX2, ZX27, and ZX69)	1. Antibacterial tests in vitro by agar spot and well diffusion tests 2. Antibacterial testing of untreated cell-free supernatant (CFS) and CFN 3. Antibiotic susceptibility testing 4. Detection of plantaricin-related genes 5. Virulence genes in *G. vaginalis* affected by *Lactobacillus*	In vitro	*G. vaginalis* (ATCC49145)	Upregulation of the transcription levels of antimicrobial resistance genes in *G. vaginalis* was demonstrated.	[101]
11	LA		1. Lactate dehydrogenase (LDH) assay 2. HIV-leakage assay 3. TNF-α ELISA 4. qRT-PCR 5. Air–liquid interface (ALI) cultures	In vitro (VK2 E6/E7 (Vk2) vaginal epithelial cell line)	*G. vaginalis*	Vaginal microbiome improved.	[91]
			1. LA determination 2. Killing activity in co-culture conditions 3. Characterization of the killing activity of Lactobacillus CF-culture CSs 4. Killing activity of LA and H_2_O_2_	In vitro	*G. vaginalis* DSM 4944	Vaginal pathogens, including *G. vaginalis*, were negatively affected by LA.	[92]
			1. Histopathological examination 2. ELISA for cytokine detection 3. qPCR 4. Vaginal microbiota analysis	In vivo (Female BALB/c)	*G. vaginalis* ATCC 14018	Significant growth inhibitions of the tested bacteria were observed.	[93]

SLD—sialidase, BV—bacterial vaginosis, CFU—colony-forming unit, HPLC—high-performance liquid chromatography.

The present study focused on *Lactobacillus* strains loaded with probiotics and their anti-*G. vaginalis* potential across BV treatment. Anin vitro study showed that maximum adherence was exhibited by DM8909 (593 ± 112) and ZX27 (209 ± 13) strains, and produced the highest amount of LA. Gene expression assay showed that all strains effectively alter *G. vaginalis* genes (*gtf*, *bcrA*, *mds*, *vly*, and *sld*), which are associated with biofilm and MDR. Authors have concluded that these probiotic mixtures enable overcoming MDR and hold promising actions [97]. Moon et al., 2022, have investigated the in vitro and in vivo anti-*G. vaginalis* potential of CH88 cell-free supernatant, and in their study, *G. vaginalis* growth and biofilm formation were significantly inhibited. Thein vivo assay exhibited significant suppression of CFU in the mice model vaginal fluid and regulated immune response. This research data highlights nutraceutical activity and hence could be used as an alternative to conventional therapy [98].

The present study demonstrated in vivo anti-*G. vaginalis* activity of three lactic acid-producing bacteria (LAB), such as *Ligilactobacillus salivarius* MG242, *Limosilactobacillus fermentum* MG901, and *Lactiplantibacillus plantarum* MG989 (Kim et al., 2022). In their study, they found that up to 43% of *G. vaginalis* growth inhibition was observed after oral administration of LAB. Moreover, vaginal epithelial tissue exfoliation reduction and pro-inflammatory downregulation were observed after exposure to LAB; hence, it could be a better alternative for ameliorating BV [99]. In another study by Pessoa et al., 2017, they investigated *Lactobacilli* strains such as *L. fermentum* 5.2, *L. plantarum* 6.2, and *L. plantarum* 7.1, which were collected from cocoa fermentation against *G. vaginalis*. Outcomes showed that all strains have significantly interfered with the growth of *G. vaginalis* by high hydrophobicity and auto-aggregation mechanisms. These insights significantly suppressed bacterial growth in vaginal microflora without demonstrating any side effects [100]. Zhao et al., 2020, studied anti-*G. vaginalis* activity of four *L. plantarum* strains (ZX1, ZX2, ZX27, and ZX69), which were collected from Mongolian yogurt. Outcomes showed that maximum antibacterial potential (13.67 ± 1.70) exhibited by the ZX27 strain and all tested strains were able to alter *G. vaginalis* virulence gene expression, which was necessary for its pathogenic transitions; hence, it could be used as an alternative in the future as it showed promising activity [101].

## 7. Protein Roles in BV Pathogenesis and Management

Preclinical studies demonstrate that proteins play a crucial role in the management of vaginal infections by modulating the host immunity and restoring vaginal microbial balance (Table 5). For instance, Pino et al., 2017, conducted an open, prospective, randomized trial in women with bacterial vaginosis and demonstrated the antibacterial activity of lactoferrin. Their results showed that vaginal lactoferrin positively modulated the vaginal microbiome by enhancing *Lactobacillus* abundance and reducing pathogenic bacteria such as *G. vaginalis* and *P. bivia*. Notably, *L. helveticus* remained a dominant species in the vaginal mucosa before and after lactoferrin therapy [102]. Alanwaret al.,2023, conducted a randomized controlled clinical trial in women with recurrent BV during the third trimester of singleton pregnancies. Their findings demonstrated that lactoferrin exerted beneficial antibacterial effects on the vaginal environment [103]. Pino et al., 2022, investigated the in vitro anti-*G. vaginalis* (MTZ resistance) potential of bovine lactoferrin (iron-binding glycoprotein), and in their study, they have noticed that lactoferrin inhibited the growth of *G. vaginalis* in a concentration-dependent manner (32, 16, 8, 4, 2, 1, and 0.5 mg/mL), including MTZ-resistant strains. Authors have also investigated the antibacterial efficacy of lactoferrin without an iron source and observed that lactoferrin still did not support *G. vaginalis* growth; hence, it could be a potential alternative treatment option [104]. The present study focused on the in vitro evaluation of Endolysin against the biofilm community of associated bacteria (Johnston et al., 2023). The authors performed an Endolysin treatment of a polymicrobial biofilm study, and they observed that the endolysin protein showed a positive impact towards suppression of *G. vaginalis* biofilm by reducing the live bacteria count. Significant reduction in *G. vaginalis* viability was noticed after endolysin exposure; hence, it could be an effective alternative option for biofilm manipulation [105].

An in vitro study by Moreno et al., 2022, targeted the biofilm of *G. vaginalis* by identifying endolysin candidates. Maximum *G. vaginalis* biofilm prevention occurred by endolysin candidates such as CCB2M94_8 and CCB7.1, and comparatively higher disruption of biofilm was observed by CCB7.1 (MIC-50 µg/mL) and CCB8.1 (MIC-200 µg/mL) endolysin. Moreover, investigators also concluded that this could be a promising future alternative innovation across recurrent BV treatment approaches, as it avoids MDR caused by antibiotics and promotes healthy vaginal flora [106]. In this present study, Luo et al., 2023, have included the National Health and Nutrition Examination Survey (NHANES), which further demonstrated a correlation between homocysteine (HCY) and prevalence. Their study showed that HCY was positively associated with BV, which further resulted in the lower serum level of HCY being efficaciously helpful towards management and prevention [107]. Alla et al., 2001, have isolated a bacteriocin from *Lactobacillus* acidophilus (vaginal strain) and evaluated itsin vitro anti-*G. vaginalis* activity. Authors have concluded that this biologically active protein efficaciously inhibited the *G. vaginalis* strain and could be used further [108]. Turovskiy et al., 2009, evaluated the anti-*G. vaginalis* efficacy of bacteriocin, which is isolated from healthy vaginal bacteria. In their study, they have investigated that bacteriocin protein was able to alter the *G. vaginalis* cytoplasmic membrane and suppress its virulence potential [109].

## 8. Fatty Acid Roles in BV Pathogenesis and Management

In several research studies, fatty acids have been found to maintain the acidic pH of the vagina, thereby supporting the growth of protective Lactobacillus species (Table 6). For example, Strandberg et al., 2010, have assessed the in vitro anti-*G. vaginalis* efficacy of the Glycerol Monolaurate (GML) fatty acid in BV patients. In their study, a ≥3-log drop was observed in common fungicidal unit (CFU/mL) for *G. vaginalis* bacteria after administration of GML for 24 h at a 10 μg/mL concentration. Authors also demonstrated that GML potentially inhibited the growth of *G. vaginalis* by stabilizing the vaginal microbiome with exhibiting anti-inflammatory action [110]. Schwecht et al., 2023, have evaluated LA potential, and in their study, results demonstrated that LA and butyric, succinic, and acetic acids have improved barrier integrity of vaginal epithelium and ameliorated inflammatory effects [111]. 

## 9. Natural Compounds and Essential Oils in BV

Constituents derived from both plant and microbial sources could be a path to promising antibacterial activity in the history of mankind [104,105,114]. In this section of this critical review, we have discussed natural molecules and their novel pattern insights in depth towards inhibiting responsible pathogens (Table 7). Fan et al., 2023, studied the in vivo antimicrobial potential of *Sophora favescens* against 30 MTZ–resistant clinical strains of *G. vaginalis* from BV patients. In their study, they have learned that, at MIC 0.3125 to 1.25 mg/mL, *S. favescens* inhibited MTZ resistance in *G. vaginalis* growth; also, at MBIC 0.625 to 1.25 mg/mL, this alkaloid has significantly eradicated bacterial biofilms. Authors have also noticed that, after exposure to *S. favescens* alkaloid, bacterial biofilm shape changed from thick to flaky, which further indicated that this herbal approach not only disrupts the bacterial biofilm but also changes its microstructure and morphology [115]. Nord et al., 2019, have first evaluated the antimicrobial activity of Spathullin A (6,7-dihydroxy-5,10-dihydropyrrolo[1,2-b] isoquinoline-3-carboxylic acid) and Spathullin B (5,10-dihydropyrrolo[1,2-b] isoquinoline-6,7-diol) against Gram-negative and -positive bacteria. Outcomes revealed that spathullin B showed higher antibacterial potential against tested strains (*E. coli* and *S. aureus*, etc.) at MIC < 1 µg/mL than the spathullin A compound. Investigators concluded that, due to some issues associated with spathullin A (stability) and spathullin B (toxicity), they could be of low interest as further medication across BV unless a suitable formulation is developed [116]. Askari et al., 2020, conducted a randomized clinical trial to evaluate the anti-BV potential of myrtle and oak gall (MOGS) and also compared its activity with MTZ and placebo. Outcomes revealed that vaginal discharge improved more after administration of MOGS (mean 0.64 ± 0.50) than MTZ (1.55 ± 0.82); moreover, clinical signs and symptoms of BV were significantly suppressed by MOGS. Hence, it might be used further for developing new treatment options [117].

Yadav et al., 2019, have performed anti-*E. coli* activity of *Azadirachta indica*, *Cichorium intybus* (leaves), and *Trigonella graecum*, and in their study, they have demonstrated that a maximum antibacterial activity of 20.7 ± 1.15 and 18.0 ± 1.0 was observed after exposure of *A. indica* water extracts and hydro alcoholic extracts of *T. foenum graecum*, and could be used in the future as an alternative [118]. Selis et al., 2021, have aimed to characterize antibacterial actions of five *Lactobacillus* strains, such as *L. casei*, *L. fermentum*, and *L. plantarum*, isolated from *Theobroma grandiflorum*. From an in vitro antimicrobial assay, it was revealed that the maximum *anti-G. vaginalis* potential (35.55 ± 2.98) and *G. vaginalis* inhibition zone (IZ) (21.00 ± 0.00 mm) exhibited by Lp90 and Lc24 *Lactobacillus*,comparatively, produced maximum H_2_O_2_. All strains produced better efficacy by stabilizing vaginal pH [119]. Essential oils have also demonstrated potent activity against vaginal infections (Table 8). For instance, Machado et al., 2017, have studied and compared in vitro anti-*G. vaginalis* activity, and in their study, they demonstrated that, at MIC 0.16 μL/mL, both *T. capitata* EO and carvacrol revealed efficacious antibacterial potential. Biofilm biomass reduction in *G. vaginalis* has been observed by both EOs and carvacrol at a 0.64 μL/mL concentration. Authors have confirmed that EOs showed more biofilm disruption activity than carvacrol; hence, it could stand out as an efficacious therapy [120]. Rosca et al., 2022, evaluated, for the first time, antimicrobial assays against polymicrobial biofilm formed by six spp. At an MIC ranging from 0.16 to 0.31 µL mL^−1^, EOs exhibited antibacterial activity against all spp., whereas MTZ showed MIC 8–32 µL mL^−1^ against *G. vaginalis*. The potential of EOs revealed that the biofilm of all spp. was significantly inhibited and stands out as an efficacious alternative [121].

Bogavac et al., 2016, have evaluated the antibacterial efficacy of *Rosmarinus officinalis* EO against *E. coli*, which is also responsible for BV development. From an in vitro antimicrobial study, it was learned that, at MIC/MFC of 12.5 mg/mL, EO revealed a promising effect and could be a futuristic alternative to synthetic medication [122]. Within theirin vitro and in vivo anti-*G. vaginalis* study, Trinh et al., 2011, investigated *Artemisia princeps Pamp.* EO (active compound: eucalyptol and α-terpineol) potential. At MIC 0.06% (*v*/*v*), growth of *G. vaginalis* was more inhibited by α-terpineol than eucalyptol; also, both of the herbal compounds significantly suppressed viable *G. vaginalis* numbers in vaginal mucosa. Authors have also noticed that both of these compounds also potentially inhibited pro-inflammatory cytokines and ameliorated BV [123]. Schwiertz et al., 2006, demonstrated the antibacterial efficacy of ten EOs (lemongrass, palmarosa, tea tree, neroli, manuka, lavender, rosemary, thyme linalool, geranium, and clary sage), and, in their in vitro study, they found that every EO was able to significantly inhibit *G. vaginalis* growth at 1–2.5 μL/mL (MIC and MBC); however, maximum *G. vaginalis* growth inhibitions were noticed by lemongrass, palmarosa, lavender, and geranium EO administration [124].

## 10. Vitamins’ Roles in BV Pathogenesis and Management

Vitamins contribute to the management of BV by demonstrating potent antimicrobial activity (Table 9). For instance, Mojtahedi et al., 2023, have conducted a case–control study to evaluate the association between 25-hydroxy vitamin D and incidence of BV for further therapeutic advancement. Key findings of their study were that lower serum levels of vitamin D were observed in BV cases, and it also exhibited an inverse association with 25-hydroxy vitamin D. The authors concluded that deficiency of this vitamin could cause/elevate incidence. However, further studies should be conducted to evaluate its potential across BV [125]. This meta-analysis study aims to assess the vitamin D deficiency and BV risk association in pregnant women [126]. Analysts have concluded that vitamin D is inversely associated with adverse health outcomes and a high incidence of BV in women. Vitamin D supplementation/intake could give a positive/effective impact on the incidence of BV, especially in pregnant women, and further studies still need to be carried out to develop an alternative treatment in the future [126].

In this cross-sectional study, Cui et al., 2023, have included NHANES in the years 2001–2004, which demonstrated an association between folate and the development and pathogenesis of BV. In their study, investigators found that serum folate has a negative/inverse association. Investigators also observed that adequate supplementation with folate could give potential antimicrobial efficacy and suppress the associated pathogenic risk, which could be a suitable alternative treatment option [127]. Alizadeh et al., 2017, evaluated the antibacterial effect of vaginal tablets containing vitamin C on the treatment and recurrence by designing a triple-blind randomized clinical trial. In their study, the intervention group was administered vitamin C with oral MTZ for 7 days, and after that, the results showed that, comparatively, maximum clinical symptoms associated were suppressed/reduced in the intervention group than in patients treated with the placebo group (Placebo + MTZ). Researchers also demonstrated that 84.8% of patients were more satisfied with vitamin C in the MTZ treatment group than with the placebo (75.6%) [128]. In another study by Krasnopolskya et al., 2013, they designed a randomized, double-blind, placebo-controlled, parallel-group clinical trial to evaluate vitamin C vaginal tablets (200 mg ascorbic acid) on recurrent episodes. In their study, investigators concluded that 6.8 and 14.7% of patients (vitamin C group) and 14.7 and 32.4% of patients (placebo group) experienced recurrence of BV after 3 and 6 months of treatment therapy. Constant use of vitamin C helped to reduce the incidence of its recurrent form, and that could be useful further as an alternative to synthetic medication [129].

## 11. Miscellaneous Factors in BV Pathogenesis and Management

Hymes et al., 2013, have studied the in vitro and in vivo anti-*G. vaginalis* potential of DNase, and in this work, the authors targeted the eDNA, which is associated with *G. vaginalis* biofilm. Outcomes revealed that DNase significantly inhibits and disrupts bacterial biofilm, and not only does it do that, but it also potentiates antibiotics’ efficacy against *G. vaginalis* biofilm. Authors also observed that DNase inhibits bacterial colonization; hence, it could stand out as an alternative therapy option [112]. Sarabia et al., 2020, demonstrated SLD inhibition of *G. vaginalis* by producing a monoclonal antibody (mAb) in MAP8 format. In this study, investigators observed that anti-SLD mAb efficiently suppressed SLD activity in *G. vaginalis* and could be used in the future by reducing complications associated with BV [113].

## 12. Herbal Nanoformulations for BV

In this section, we analyzed nanotechnology-based delivery systems that have brought numerous advances to improve the therapeutic properties of herbal drugs. These systems offer technical optimizations, such as sustained, controlled, and targeted drug release, which enhance bioavailability, improve site-specific delivery, and reduce systemic side effects for more effective patient-specific applications [25,130,131]. Integration of natural source constituents with nanomaterials overcomes drawbacks associated with herbal drugs, like stability, compatibility, etc. This technology reframes the therapeutic properties by improving its bioavailability. Table 10 outlines a detailed compilation of novel formulations comprising natural-based constituents used in BV.

Kaczmarek et al., 2022, demonstrated that a chitosan-based system with *Scutellariae baicalensis radix* (SBE) extracts acts as a potential implementation towards vaginal infections like BV. In their in vitro antimicrobial assay, they have compared the potential of SBE, SBE-loaded Cs, MTZ, and clindamycin with each other to evaluate therapeutic efficacy. They further observed that, at MIC-800 µg mL^−1^ and a ratio of 2:1, SBE (27.0 ± 2.0) and SBE/Cs (25.0 ± 2.0) exhibited a better maximum IZ than the marketed medication (MTZ and clindamycin), which were tested at MIC 1000 µg mL^−1^ against *G. vaginalis*. They have also concluded that an herbal extract-loaded chitosan system could be a better alternative in the future for vaginal drug delivery, as it refines the vaginal microbiome by reducing inflammation [132]. A mucoadhesive chitosan tablet system containing *Chelidonii herba* extracts has been formulated by Paczkowska et al., 2020, to overcome vaginal bacterial infection. Findings showed that, out of all formulations, F3 with HPMC in it exhibited maximum mucus adhesion in porcine vaginal mucosa. From this study, it was concluded that this formulation could be a good alternative candidate across BV (Table 10), as it has shown potential antibacterial efficacy with considerable inflammation [133].

Arrue et al., 2023 formulated biosurfactants from probiotic *Bacillus* spp. at concentrations such as 1% (BNE1%) and 3.33 wt% (BNE3.33%) in a nanoemulsion system and evaluated their antibacterial potential against E. coli, which is a potential spp. This helps in developing BV. The results showed that probiotic-based BNE3.33% nanoemulsion was potentially able to inhibit bacterial adhesion and disrupt bacterial cell integrity. The BNE3.33% formulation exhibited a low MIC of 0.5 mg/mL, demonstrating effective antimicrobial activity compared to all other formulations [134]. Bouaouina et al., 2022 have evaluated the antimicrobial action of *Origanum glandulosum Desf* by encapsulating it into a nanoemulsion-based system. In Vitro antibacterial assay showed that, at MIC-0.62%, *O. glandulosum* oil exhibited an efficacious IZ of 13 mm, whereas herbal-based nanocapsules and nanoemulsions showed IZ of 11 and 20 mm against MDR bacteria spp. Authors also learned that nanoemulsion potentially inhibited bacterial biofilm than nanocapsule, which could be used further for an alternative treatment option [135]. Tomás et al., 2023 have incorporated *Thymbra capitata* EO (TCEO) into a suitable vaginal sheet system to evaluate its anti-*G. vaginalis* potential. A cellular toxicity study demonstrated that, at adequate concentration, the herbal vaginal sheet revealed nontoxic action to the HEC 1A cell line, and an antimicrobial assay revealed that the TCEO vaginal sheet was significantly able to inhibit *G. vaginalis* load and its biofilm at 0.32 µL/mL concentration. At last, researchers concluded that these herbal-based vaginal sheets significantly inhibited *G. vaginalis* with dose-dependent concentration [136].

## 13. Underlying Mechanisms of BV

**Figure 4:**
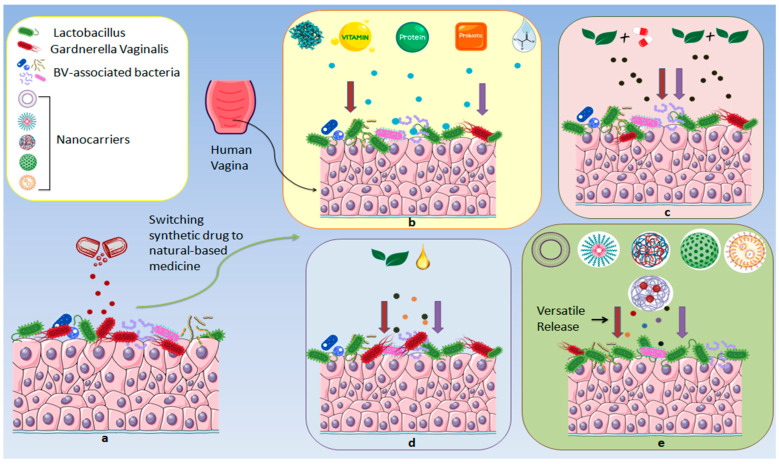
Comparative antibacterial potential between current synthetic medication and constituents derived from a natural source. (**a**) Depicts the therapeutic efficacy of marketed antibiotics, which led to less antibacterial activity due to resistance. (**b**–**d**) Depicts comparatively higher anti-*G. vaginalis* activity. (**e**) Depicts maximum antibacterial potential due to the integration of nanomaterials with natural source constituents, which exhibited higher antibacterial characteristics. Generated by Biorender (2025) https://app.biorender.com/, [Date accessed: 12 March 2025].

**Figure 5:**
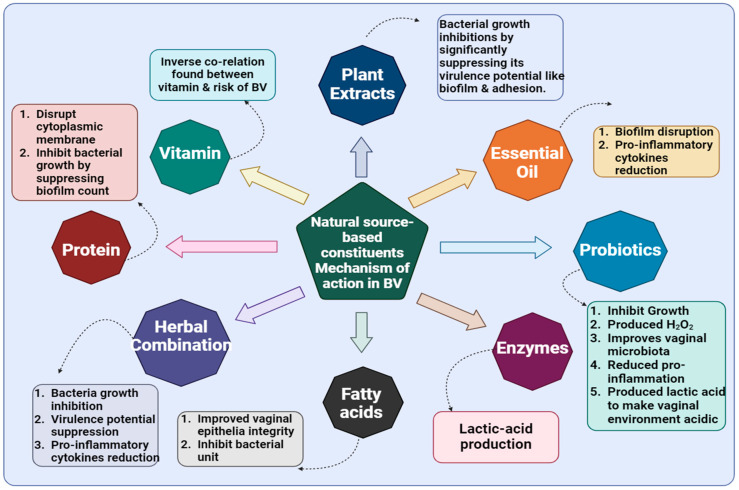
A schematic illustration concerning the mechanism of natural source-based constituents against pathogenic bacteria involved in inducing BV. Generated by Biorender (2025) https://app.biorender.com/, [Date accessed: 10 March 2025].

In this section, we have critically discussed novel mechanistic insights regarding herbal molecules in BV while minimizing MDR [137,138]. Natural molecules, nutraceuticals, vitamins, enzymes, proteins, plant-derived compounds, EOs, and fatty acids exhibit broad mechanistic insights by targeting dysbiosis and biofilm. A healthy vagina is a *Lactobacillus*-dominated microbiota that prevents overgrowth of anaerobic bacteria, i.e., *G. vaginalis*, which exhibits pathogenic transition and leads to virulence factors (Figure 1). Nutraceuticals and plant-derived molecules can disrupt adhesion and biofilm integrity through interference with quorum sensing, key enzymes, and modulation of host cell surface molecules, potentially restoring a vaginal microbiome. Vitamins, especially vitamin C, may lower vaginal pH and support epithelial barrier function, indirectly supporting *Lactobacillus* growth. Enzymes such as DNases or proteases can degrade ECM in biofilms, enhancing the susceptibility of pathogenic bacteria to host immunity and antimicrobial agents. Proteins can directly lyse bacterial membranes or modulate immune responses, enhancing the clearance of BVAB. EOs and fatty acids disrupt *G. vaginalis* membranes and inhibit biofilm formation. Nanomaterials, such as Au or chitosan NPs, offer targeted delivery and enhanced site-specific penetration, enabling antimicrobial action or delivery of bioactive compounds while protecting the healthy microbiome (Figure 4). Additionally, these molecules act through diverse mechanisms (Figure 5), modulating immune responses and directly killing or suppressing BVAB without observed toxicity, offering promising alternatives to conventional therapies, especially in the context of biofilm-associated resistance (Figure 3) and recurrent episodes.

## 14. Critical Analysis of Patent Literature on BV

Patent data plays a vital role for investigators to focus more on naturally based constituents loaded into novel formulations, which might gain attention as an alternative to conventional therapies in terms of potential, MDR, and recurrence rate [139], summarizing a comprehensive patent showcasing a diverse and evolving therapeutic arsenal targeting BV, encompassing plant-based interventions, EOs, vitamins, probiotics, proteins, enzymes, and NPs-based systems (Table 11). Plant-derived formulations, particularly from traditional Chinese medicine and herbal combinations, exhibited anti-inflammatory and biofilm inhibitory properties with no toxicity noticed. This evident research data holds immense potential and might stand as an alternative. Several entries emphasize the effectiveness of epigallocatechin gallate and traditional blends in suppository or douche forms, with claims of suppressed recurrence and hormonal balance restoration. EOs have been explored for their antimicrobial and anti-inflammatory efficacy, showing promising results like microbial clearance and symptom reduction without irritation. Notable innovations in the probiotic sector have been observed, such as targeting BV directly but also restoring vaginal flora and maintaining vaginal pH using strains like *L. crispatus*, *L. acidophilus*, and *Bifidobacterium* spp. Investigators also integrate folic acid or employ delivery innovations such as layered condoms and effervescent vaginal tablets for sustained microbial balance and infection prevention. Vitamin-based supplements, especially ascorbic acid, vitamin E, and complex B, often in combination with probiotics or plant extracts, showed enhanced mucosal immunity, restored tissue health, and supported microbiome recovery. Moreover, protein-based interventions, like CXCL12, are outlined in immunological modulation alongside microbial targeting. Collectively, these patents summarize a necessary shift toward multi-mechanistic, biocompatible, and microbiota-supportive interventions. The incorporation of natural molecules with targeted delivery systems addresses MDR and recurrence.

## 15. Conclusions

This review highlights the promising potential of natural bioactive compounds, such as berberine, curcumin, thymol, allicin, and TTO, as adjunct or alternative therapies for *G. vaginalis*-induced BV. These phytoconstituents exhibit strong antimicrobial, anti-inflammatory, and immunomodulatory effects, selectively inhibiting BV pathogens while supporting beneficial *Lactobacillus* spp. Clinical evidence, including a randomized trial, shows curcumin to be as effective, if not more so, than MTZ in treating BV, with fewer side effects and an 82% cure rate versus 42% for MTZ two weeks post treatment. However, the clinical utility of these compounds is constrained by poor solubility, instability in vaginal environments, and rapid systemic clearance. NP-based delivery systems, such as liposomes, solid lipid NPs, polymeric NPs, and vaginal nanogels, address these challenges by enhancing solubility, stability, targeted delivery, mucoadhesion, and controlled release. Safety, manufacturing scalability, regulatory compliance, and patient acceptability, particularly concerning intravaginal use, must be thoroughly investigated. Additionally, exploration of synergistic formulations combining nanoherbal agents with probiotics (e.g., *L. crispatus* LACTIN V) is notably lacking, even though live biotherapeutics show promise in reducing BV recurrence. The future of BV management lies in a personalized, multi-targeted nanomedicine approach. Integrating robust phytocompounds with advanced delivery technologies, alongside precision microbiome-directed strategies, could revolutionize treatment by maximizing efficacy and minimizing resistance and harm. Finally, harnessing the synergistic power of natural molecules and nanotechnology offers a multifaceted, next-generation therapeutic paradigm for BV, one that could overcome current limitations and usher in safer, more effective, and patient-friendly treatments.

## Figures and Tables

**Table 1:** Some of the current medicines for BV.

No.	Formulation	Dose	Administration	Company Name (Example)	Typical Indication/Organisms	Adverse Effects	Pregnancy Note	References
1	MTZ (metronidazole)	500 mg	Orally (twice daily for a week)	Pfizer (Pfizer Inc., New York, NY, USA)	BV; anaerobes; *T. vaginalis* (alternative regimens)	Difficulty in breathing, stomach pain, headaches, back pain, blurred vision.	Commonly used in pregnancy when indicated (discuss in text and follow local guidance).	[52]
2	Clindamycin (cream 2.0%)	2.0% cream	Intravaginally (once daily for a week)	Alkem laboratories (Alkem Laboratories Ltd., Mumbai, India)	BV (topical/local therapy)	Redness, burning, itching at the site of application. Stomach pain or cramping.	Topical use generally considered when needed; discussed in text (see pregnancy studies).	[53]
3	MTZ (0.75% gel)	0.75% gel	Intravaginally (once daily for a week)	Galderma laboratories (Galderma Laboratories, LP, Dallas, TX, USA)	BV; local therapy for vaginal anaerobes	Vaginal itching, redness, vaginal discharge may occur.	Topical therapy often used in pregnancy as alternative—discussed evidence in text.	[54]
4	Clindamycin (oral)	300 mg	Orally (twice daily for a week)	Pfizer	BV (oral alternative), skin/soft-tissue infections (depending on context)	Abdominal pain, heartburn, diarrhea, nausea, and vomiting.	Oral clindamycin used in pregnancy in some situations; weigh benefits/risks.	[55]
5	Tinidazole	1 g	Orally (once daily for 2 days)	Farlex Pharmaceuticals Pvt Ltd. (Farlex Pharmaceuticals Pvt. Ltd., Panchkula, India)	*T. vaginalis*, protozoa (also used in BV in some regimens)	Headache, constipation, dizziness, stomach pain, cramps.	Generally avoid in pregnancy unless necessary; check label.	[56]
6	Clindamycin (ovule)	100 mg	Intravaginally (once daily for 3 days)	Pfizer	BV (intravaginal ovule)	Redness, burning, itching at the site of application.	Topical intravaginal clindamycin studied in pregnancy (see Joesoef et al.).	[57]
7	Tinidazole	2 g	Orally (once daily for 5 days)	Pfizer	*T. vaginalis*; protozoal infections	Upset stomach, nausea, vomiting, diarrhea.	As above (avoid in pregnancy unless indicated).	[54]
8	Metronidazole (single-dose option)	2 g	Oral—single dose (alternative to 7-day regimen for *T. vaginalis*)	Generic (Flagyl^®^) Pfizer Inc. New York, NY, USA	*T. vaginalis* (single-dose therapy possible); BV alternative regimens	Metallic taste, nausea, disulfiram-like reaction with alcohol, GI upset.	Metronidazole has pregnancy data; use per guideline and local practice.	[54,58]
9	Secnidazole (oral granules—single dose)	2 g	Single oral packet (sprinkle on food)	SOLOSEC^®^ Lupin Pharmaceuticals, Inc., Baltimore, MD, USA	Single-dose option for BV; also indicated for trichomoniasis in some labels	Nausea; vulvovaginal candidiasis; alcohol interaction advice on label	Check product label and regional approvals for pregnancy guidance.	[54,59]
10	Metronidazole (IV)	500 mg	IV q8 h (or q6–8 h per severity)	Generic hospital IV formulation	Severe/complicated anaerobic infections, intra-abdominal sepsis, severe gynecologic infections	Same systemic effects as oral; IV-local infusion reactions; neurotoxicity with prolonged use	Use in pregnancy only if clinically indicated; follow obstetric/infectious-disease guidance.	[58,60]
11	Clindamycin (IV)	600–900 mg	IV q6–8 h depending on diagnosis	Cleocin^®^ Pfizer Inc.—New York, NY, USA	Severe pelvic infections, necrotizing soft-tissue infection, alternative for anaerobes	Diarrhea, risk of C. difficile colitis, hypersensitivity	Clindamycin used in pregnancy in certain contexts (e.g., gynecologic infections)—see refs.	[60,61]
12	Vancomycin (oral)	125 mg	Oral q6 h × 10 days	Vancocin^®^ Capsules (U.S.) ANI Pharmaceuticals, Inc. Baudette, MN, USA	First-line for initial Clostridioides difficile infection (PO)	Local GI effects; poor systemic absorption (benefit for GI infection)	Use in pregnancy per guideline (benefit > risk for severe CDI).	[60]
13	Fidaxomicin (oral)	200 mg	Oral BID × 10 days	Dificid^®^ (fidaxomicin) E. Lincoln Avenue, Rahway, NJ, USA	Alternative/often preferred agent for initial C. difficile (lower recurrence)	Nausea, GI upset; higher cost/limited availability	Considered acceptable in pregnancy when indicated (consult local guidance).	[60,62]
14	Clindamycin (vaginal ovule/supp.)	100 mg	Intravaginally at bedtime × 3 days	Cleocin^®^ Vaginal Ovules. Pharmacia & Upjohn Company LLC, New York, NY, USA	BV; local therapy	Local burning/irritation; oleaginous base can weaken latex condoms	Label/clinical notes recommend condom caution; pregnancy evidence discussed separately.	[57]

Note: Dosing shown is typical adult dosing; local/regional guidelines and product labels should be consulted. Pregnancy/lactation notes are summary statements, and administration requires consultation with obstetric guidance and product labeling.

**Table 3:** Enlists a comparison of clinical practices between constituents from natural sources and conventional ones concerning *G. vaginalis*.

Sl. No.	Comparison	Type of Study	Trial Registration No.	No. of Patients	Dose	Observation	References
1.	Sucrose gel with MTZ gel	Triple-blind, parallel, randomized clinical trial	IRCT2016112631105N1	70	MTZ gel 0.75% and Sucrose gel 9%	Sucrose vaginal gel comparatively improved vaginal microflora.	[77]
2.	Kakrasingi with MTZ	Randomized (1:1), standard controlled, single-center	100609	62	Kakrasingi (1 g) and MTZ (400 mg)	No side effects were demonstrated in herbal-based medicine.	[78]
3.	Quercus (Oak Gal) cream with MTZ gel	Double-blind, randomized controlled trial	IRCT20180122038477N1	84	Oak Gall vaginal cream and MTZ vaginal gel (5 g)	Promising antibacterial activity was demonstrated.	[79]
4.	*Quercus brantiilindl* vaginal cream with placebo and MTZ tablet	Randomized clinical trial	IRCT2016071428929N1	176	*Q. brantii* and MTZ (500 mg)	Clinical symptoms of BV were suppressed after herbal cream treatment.	[80]
5.	*Calendulae* extractumoleosum, *Bursae pastoris* extractumoleosum, *Matricariae* extractumoleosum, *Hyperici* extractumoleosum, and *Millefolii* extractumoleosum withtea tree oil (TTO)	Randomized controlled clinical, in vitro	NCT04558697	210	TTO (200 mg)	The herbal treatment showed potential anti-*G. vaginalis* activity.	[81]
6.	*Myrtus communis* L. and *Berberis vulgaris* with MTZ vaginal gel	Randomized clinical trial, in vitro and in vivo	IRCT201411102085N13	120	*M. communis* (77.1 ug/mL), *B. vulgaris* (157 mg/g)	Desired antibacterial and anti-inflammatory properties were observed.	[82]
7.	*Berberis vulgaris* gel with MTZ gel	Double-blind clinical trial	IRCT201411102085N13	80	*B. vulgaris* gel 5% and MTZ gel 0.75%	Herbal-based vaginal gel exhibited higher effective activity across BV.	[83]
8.	Garlic tablet with oral MTZ	Single-blind randomized controlled clinical trial	IRCT201207153226N4	120	Garlic tablet (500 mg) and MTZ tablet (250 mg)	Fewer side effects were observed in the garlic group of patients.	[84]
9.	Probiotics with MTZ	Randomized clinical trial Triple-blind randomized clinical trial	NCT02459665 215121721917N5	68 100	MTZ (500 mg), prebiotic vaginal gel (5 mg), and MTZ (250 mg)	Beneficial antimicrobial efficacy was observed in probiotics. Probiotic vaginal gel improved clinical symptoms of BV and refined the vaginal microbiome.	[76]

MTZ—Metronidazole, BV—Bacterial Vaginosis, TTO—Tea Tree Oil.

**Table 5:** Therapeutic Role of Proteins in the Management of BV.

Sl. No.	Proteins Used	Types of Performed Study	Experimental Types	Antibacterial Against	Key Findings	References
1.	Lactoferrin	1. Microbiological analysis 2. RNA isolation from vaginal swabs 3. Ion Torrent 16S rRNA gene-based analysis 4. Taxonomic identification	Open prospective randomized trial (SHI-EVE-2014.01)	Sixty women with BV	Improvement of the vaginal ecosystem was observed after lactoferrin administration.	[102]
		1. Antibacterial study	Randomized controlled clinical trial	66 cases with a history of recurrent BV	Beneficial effects were investigated.	[103]
	Bovine Lactoferrin (MTbLF)	1. Antimicrobial susceptibility testing 2. Antimicrobial activity of bovine lactoferrin against presumptive *G. vaginalis* clinical isolates 3. Antibiotic interference/synergy test	In vitro antimicrobial study, Clinical trial (ACTRN12619000295145)	*G. vaginalis* clinical isolates	Promising anti-*G. vaginalis* efficacy was demonstrated.	[104]
2.	Endolysin	1. Monospecies biofilm formation and treatment 2. Polymicrobial biofilm treatment 3. DNA extraction and PMAxx treatment 4. SEM	In vitro	*G.vaginalis* (ATCC 14018)	Suppression of bacterial load was observed.	[105]
		1. Endolysin stability assays 2. MIC assays 3. Resistance assays using serial passages 4. Biofilm prevention and disruption assays 5. Reproducibility and statistical analysis	In vitro	*G. vaginalis* UG860107 and *A. vaginae* UG71161	Maximum biofilm inhibitions were observed by CCB2M94_8 and CCB7.1 (best endolysin candidate).	[106]
3.	Homocysteine	1. Antibacterial study	Association study	*G. vaginalis*	*G. vaginalis* growth was inhibited significantly.	[107]
4.	Bacteriocin	1. Demonstration of antimicrobial activity	In vitro	*G. vaginalis*	The desired antibacterial activity of this protein was investigated.	[108]
	Lactocin 160	1. The Membrane Disruption (Ethidium Bromide) Assay 2. The ATP Assay 3. ΔpH Dissipation Assay 4. ΔΨ Dissipation Assay	In vitro	*G. vaginalis* ATCC 14018 and *L. rhamnosus* 160	The cytoplasmic membrane of *G. vaginalis* was altered.	[109]

BV—Bacterial Vaginosis, MIC—Minimum Inhibitory Concentration.

**Table 6:** Fatty Acids Contributing to Vaginal Microbiota Stability and BV Control.

Sl. No.	Fatty Acids Used	Types of Performed Study	Experimental Types	Antibacterial Against	Key Findings	References
1.	GML	1. Antimicrobial assay	In vitro experiments and a single-center, double-blind, randomized study	Clinical isolate of *G. vaginalis*	Significant reductions were seen in *G. vaginalis* counts	[110]
**Sl. No.**	**ConstituentsUsed**	**Types of Performed Study**	**Experimental Types**	**Pathogen**	**Key Findings**	**References**
1.	DNase (Enzyme)	1. Biofilm Assays	In vitro and in vivo (eight-week-old female C57BL/6J mice)		This enzyme potentially inhibits bacterial biofilm.	[112]
2.	Monoclonal Antibody	1. *G. vaginalis* culture and growth kinetics 2. SLD production during the growth of *G. vaginalis*	In vitro (HeLa cells)	*G. vaginalis* ATCC 14018	The virulence potential of the tested pathogens was significantly suppressed.	[113]

GML—Glyceryl Monolaurate.

**Table 7:** Therapeutic potential of microorganisms and plants in the management of BV.

Sl. No.	Used Constituents	Active Molecules	Souce	Types of Performed Study	Experimental Types	Antibacterial Against	Key Findings	References
1.	*Sophora favescens* (alkaloid)	Matrine and oxymatrine	Plant	1. HPLC 2.Antimicrobial Susceptibility Testing 3. Bacterial Biofilm Formation Assay	In vitro	*G. vaginalis*. *B. fragilis* ATCC 25285	Significant bacterial growth inhibitions were observed at MIC 0.3125–1.25 mg/mL.	[115]
2.	*Penicillium spathulatum* Em19	6,7-dihydroxy-5,10-dihydropyrrolo[1,2-b]isoquinoline-3-carboxylic acid and 5,10-dihydropyrrolo[1,2-b]isoquinoline-6,7-diol	Isoquinolinealkaloids from the fungus *Penicillium Spathulatum* Em19	1. MIC Determination 2. Determination of Toxicity Huh 7 Cells (Hepatocellular Carcinoma Cells)	In vitro bioassay	*E. coli* LMG15862	The MIC range between 0.05 and 50 µg/mL was found.	[116]
3.	*Myrtus communis* L. (myrtle) and *Quercus infectoria* G. Olivier (oak gall)	Citric acid, gallotannicacid, gallic acid, andellagic acid	Plant	1. Naranjo Scale for Adverse Drug Reaction (ADR) 2. Antibacterial Study	Parallel randomized clinical trial (IRCT2016030526917N1)	120 women with vaginitis	Desired antibacterial effects were demonstrated.	[117]
4.	*Azadirachta indica* (AI), *Cichorium intybus* (CI), and *Trigonella foenum-graecum* (TFG)	beta.dMannofuranoside and O-geranyl	Plant	1. Antimicrobial Screening Using Agar Well Diffusion Method	In vitro	*E. coli* (Strain No. 8739-8/03/15)	Broad-spectrum antimicrobial activities were investigated.	[118]
5.	*Theobroma grandiflorum* (Cupuaçu)	Linoleic acid and oleic acid	Plant (fruit)	1. Heat Tolerance Assay 2. pH Tolerance Assay 3. Antibiotic Susceptibility Assay 4. Hemolytic Activity Assay 5. Biofilm Formation Assay 6. Hydrophobicity Assay 7. Auto-aggregation Assay 8. Co-aggregation Assay 9. Inhibition Assay by Coculture	In vitro	*G. vaginalis* ATCC 49154	Vaginal microbiota improved.	[119]

**Table 8:** Antimicrobial Activity of EOs Against Pathogens Involved in BV.

Sl. No.	EO Used	Active Molecules	Types of Performed Study	Experimental Types	Antibacterial Against	Key Findings	References
1.	*Thymbra capitata* EO	Carvacrol and thymol	1. MIC and minimal lethal concentration determination 2. Flow cytometry analysis 3. Biofilm formation and quantification 4. Confocal laser scanning microscopy analysis of *G. vaginalis* biofilms	In vitro	*G. vaginalis*	Bacterial biofilm was potentially inhibited.	[70]
			1. MIC and MLC determination 2. In vitro biofilm formation and quantification 3. Formation and characterization of polymicrobial biofilms on the reconstructed human vaginal epithelium 4. Characterization of in vitro and ex vivo polymicrobial biofilms	In vitro and ex vivo (vaginal epithelial tissue)	*G. vaginalis* ATCC 14018	High antibacterial effect against polymicrobial biofilms of the tested bacteria was observed.	[71]
2.	*Rosmarinus officinalis* EO	Tricyclene, α-pinene, camphene	1. Antimicrobial assay 2. Brine shrimp toxicity assay	In vitro	*E. coli* I, *E. coli* II	Significant suppression of bacterial load was demonstrated.	[122]
3.	*Artemisia princeps Pamp*. EO	Eucalyptol and *α*-terpineol	1. Antimicrobial activity assay 2. Histopathologic examinations 3. Assay of MPO activity 4. ELISA and immunoblot analysis	In vitro and in vivo (Male and female ICR mice)	*G. vaginalis* KCTC5096	No cytotoxicity effect was noticed in the murine model.	[123]
4.	*Lemongrass*, *Palmarosa*, *Tea tree*, *Neroli*, *Manuka*, *Lavender*, *Rosemary*, *Thyme linalool*, *Geranium* and *Clary sage* EO	Geranial, terpinen-4-ol, Linalool, Leptospermone, 1,8-cineole, Citronellol, myrcene	1. MIC, MBC/MFC 2. Antibacterial assay	In vitro	*G. vaginalis*	Restoration of the natural vaginal flora after exposure to EO.	[124]

MIC—Minimum Inhibitory Concentration, MPO—Myeloperoxidase, EO—Essential Oil, MBC—Minimum Bactericidal Concentration, MFC—Minimum Fungicidal Concentration.

**Table 9:** Vitamin Based Interventions and Their Antimicrobial Impact on BV.

Sl. No.	Vitamins Used	Types of Performed Study	Experimental Types	Antibacterial Against	Key Findings	References
1.	25-Hydroxy vitamin D	1. Diagnosis of BV 2. Evaluation of serum 25-hydroxy vitamin D 3. Antibacterial study	Case–control study (IR.GMU.REC.1398.150)	250 confirmed BV cases	The inverse association between vitamin and BV was investigated.	[125]
2.	Vitamin D	1. Antibacterial study	Observation study	Patients with BV	Vitamin D supplementation exhibited effective actions.	[126]
3.	Folate	1. Determination of serum folate concentrations 2. Determination of RBC folate concentrations 3. Antibacterial assay	Cross-sectional study	Patients diagnosed with BV	Serum folate was inversely associated with the risk of BV.	[127]
4.	Vitamin C	1. Antibacterial study	Triple-blind randomized clinical trial (IRCT2015042521917N3)	160 women with BV	Vaginal environment recovery was demonstrated.	[128]
		1. Antimicrobial study	Randomized, double blind, placebo-controlled, parallel-group clinical trial	144 Patients with BV	A lower recurrence risk of BV was found.	[129]

**Table 10:** Enlists the efficacy of herbal-basednanoformulations against BV.

Sl. No.	Natural-Based Constituents	Nanoformulation	No. of Performed Studies	Experimental Types	Outcomes	References
1.	SBE	Chitosan-based system	1. ATR FTIR and DFT 2. XRPD, Thermogravimetric Analysis (TG), DSC 3. Anti-Hyaluronidase Activity 4. Ferrous Ion Chelating Activity	In Vitro	Promising antibacterial activity was observed.	[132]
2.	*Chelidonii herba*	Mucoadhesive chitosan system	1. MTT Test 2. Permeability Studies 3. Microbiological Activity	In Vitro Release Studies and Ex Vivo Mucoadhesive Properties (Porcine Vaginal Mucosa)	Enhanced bioavailability observed in the chitosan system.	[133]
3.	Probiotics	Nanoemulsion	1. Antimicrobial Susceptibility Assay 2. MIC and MBC 3. Antibiofilm Assay 4. Cell Surface Hydrophobicity 5. Bacterial Cell Membrane Disintegration Test	In Vitro	Potential disintegration of the bacterial cell was observed.	[134]
4.	*Origanum glandulosum Desf*	Nanoemulsion system	1. Antibiotic Susceptibility Test 2. Broth Microdilution Assay and Agar Diffusion Method 3. Antimicrobial Assay	In Vitro	This formulation could be an alternative in BV treatment options.	[135]
5.	*Thymbra capitata* EO	Vaginal sheets	1. Bioadhesion 2. Cellular Toxicity 3. Cytotoxicity Test (MTT Assay) 4. Vaginal Irritation—Hen’s Egg TestChorioallantoic Membrane Assay (HET CAM)	HeLa and HEC 1A Cell Lines Were Used	Efficient antibacterial activity observed at 0.03 g/mL.	[136]

ATR FTIR—Attenuated total reflectance Fourier transform infrared spectroscopy, DSC—differential scanning calorimetry, MIC—minimum inhibitory concentration, MBC—minimum bactericidal concentration, EO—essential oil.

**Table 11:** Patent records of plant, essential oil (EO), vitamin, protein, probiotic, enzyme, and nanoformulationin BV.

Sl. No	Inventors	Application No.	Title of Inventive Appellation	Plant Used	Country	Publication Date	Outcomes	References
Plants								
1	Wegener et al.	WO/2006/047590	Compositions and treatments ofBV.	*Epigallocatechin gallate*, *epicatechin gallate*, epigallocatechin, epicatechin	US Patent	4 May 2006	With the help of plant polyphenols, treatment was provided for BV.	[140]
2	Xiaoyan et al.	201410265286.6	Traditional Chinese medicine for treatingBV and the preparation methods of traditional Chinese medicine.	Osbeckia herb, horseweed herb, hairy euphorbia, wandering jew, Amaranthus viridis, wideleafwideleafosbeckia root, herb of sword brake, herba *Fibraureae recisae*, *Dictamni cortex*, *Lantana camara*, *Cryptotaenia japonica hassk*, *Senecio dianthus franch*	China Patent	28 April 2005	A suppository of traditional Chinese medicine was administered once a day continuously for 15 days. No recurrence was seen while following up for 1 year.	[141]
3	Wang Xinsuo	200710120906.7	Chinese traditional medicine preparations for treatingBV.	Anemarrhena rhizome, Amur corktree bark, radix *Rehmanniae preparata*, common yam rhizome	China Patent	30 January 2008	The present intervention was only able to treat BV, but it was also able to prevent bacterial infection, had no toxicity or side effects, and had better patient compliance.	[142]
4	Nash et al.	WO/2020/115475	Bioactive phytochemicals in zizyphus and guarana	*Cucurbitaceae* extract	Indian Patent	11 June 2020	It can adhere to biofilm and has less salidase activity.	[143]
5	Soleck et al.	PCT/CA2019/050451	Cannabis root extract, method of manufacture, and method of use.	Cannabis root	American Patent	17 October 2019	Therapeutic efficacy against BV with fewer side effects.	[144]
6	Xu Chonglan	102016000121541	Chinese herbal preparation for treating BV and the preparation method.	Roots of Chinese pulsatilla, broom cypress fruits, seeds of feather cockscomb, small fruit fig aerial roots, all grass of *Pilea japonica* (maxim.)	China Patent	29 June 2016	The preparation method of the traditional Chinese method is appropriate in compatibility, can reduce inflammation, and has no adverse or toxic effects.	[145]
7.	Aдиcoвнa et al.	2009110700/14	Method of treating BV.	Herbal tincture containing camomileflowers, pine buds, birch buds, celandine herb, bird cherry, oak bark, and alder collective fruits at an equal ratio, dosed 250.0 mL	Russia Patent	10 July 2010	This method has a very good therapeutic effect on vaginal dysbiosis and helps in restoring hormonal imbalance.	[146]
8.	Jung, So Young	1020200095649	Herbal medicine composition for treating vaginitis and preventing uterine cervical dysplasia.	*Coix lachrymajobi* var. *Mayuen*, *Atracty lodeslancea*, *Dictamnus dasycarpus*, and *Sophora flavescens*	Korea Patent	5 February 2021	This herbal combination of medicine has an efficacy of preventing cervical dysplasia by effective treatment and prevention of BV and candida vaginitis.	[147]
9.	Eвгeньeвич et al.	2012126560/15	Agent for vaginal douche in phase of vaginal microflora recovery following treatment ofBVand vaginal thrush (vaginal yeast).	*Anemarrhena rhizome*, Amur corktree bark, radix *Rehmanniae preparata*, common yam rhizome	RussianPatent	10 July 2013	Use of an aqueous solution of vaginal douche creates favorable conditions for the therapeutic efficacy of medicinal herbs, which decreases the chances of developing any allergic reaction.	[148]
10.	Peian et al.	201310618673.9	Anti-inflammatory leukorrhea, arresting washing liquor.	Climbing groundsel herb, Folium isatidis, mint, wild chrysanthemum flower, and Cortex dictamni	China Patent	3 June 2015	Prepared formulation has all the natural ingredients, has no toxicity or side effects, no irritation, and also reduces inflammation.	[149]
11.	Eвгeньeвич et al.	2012126562/15	Agent for vaginal douche in first stage of treatment ofBV.	*Matricaria chamomilla* L.	RussianPatent	10 July 2013	Duration of treatment was reduced with the help of the douching procedure, and restoration of natural vaginal flora has high therapeutic efficacy.	[150]
**Essential Oil (EO) and Oil**								
				**EO and Natural Oil Used**				
1.	Luan Xiaoming	102014000533282	Vagina cleaning sanitary lotion.	Rosemary, menthol EO	China Patent	20 April 2016	Prepared formulation has a cleaning effect on HPV, fungi, and cocci and has a therapeutic efficacy against BV.	[151]
2.	Alberto et al.	08009684	Topical compositions for prevention and treatment of inflammatory and/or infective conditions of genital area.	*Matricaria chamomilla* and *Melaleuca alternifolia* EO	European Patent	14 January 2009	Recurrence of genital infections like BV can be prevented with the help of topical herbal formulations.	[152]
3.	Papa et al.	17827504	Natural composition for use in gynecology.	Olive oil	USAPatent	17 November 2022	Total negativizationof the vaginal swab was observed at the end of treatment, and relapse was only observed in 2 patients out of 10.	[153]
4.	Feifei et al.	201610664574.8	Medicinal composition for repairing female vaginal microecological balance.	TTO	China Patent	16 November 2016	Growth of probiotics was present, but growth of harmful bacteria was not present due to a lack of food.	[154]
5.	Gang et al.	202311350774.2	Vaginal antibacterial composition, private part nursing product, and preparation method, and application of private part antibacterial composition and private part nursing product.	Rape oil amide propyl dimethylamine, aspartic acid, and valerian extract	China Patent	24 November 2023	Herbal extracts have antibacterial, antifungal, and anti-inflammatory effects and can be used for the treatment of BV.	[155]
6.	Gupta Sunil	202141047207	Neem seed oil-based vaginal capsule for treatment of abnormal vaginal discharge.	Neem seed oil	Indian Patent	26 November 2021	It has 93.33% efficacy against BV and has no toxic effects.	[156]
**Probiotics**								
				**Strains Used**				
1.	Bиктopoвичet al.	2022121049	Method of treatment of BV.	*L. acidophilus* isolated from Laktonorm, Ecofemin, Gynoflor, and one strain of *L. casei* subsp. *Rhamnosus* Lcr35 isolated from Laktozhina	Russia Patent	30 September 2022	Current drug intervention has high therapeutic efficacy against BV.	[157]
2.	Komorowski et al.	17958310	Probiotic and folic acid compositions and methods of use.	*Lactobacillus* and folic acid	USAPatent	4 April 2024	The current treatment has been proven to be efficacious in clinical trials. Evaluation treatment was also proven to be safe.	[158]
3.	Kort et al.	16628762	Probioticcomposition for prevention ofBV.	Probiotics	USA Patent	7 May 2020	A total of 25 strains were tested for glycogen degradation, of which 5 strains were less efficient in degrading glycogen, and one strain did not show glycogen degradation at all.	[159]
4.	Kort et al.	WO/2019/013637	New probiotic composition for prevention of BV.	*Lactobacillus crispatus*	Netherland Patent	17.01.2019	Five strains were less efficient in glycogen degradation, and one strain was not able to degrade glycogen at all.	[160]
5.	Kompella et al.	WO/2014/020613	Probiotic-layered condom.	LA	Indian Patent	6 February 2014	Maintenance of a normal pH range for the vagina to maintain normal vaginal flora to prevent BV and other urogenital infections in females of reproductive age.	[161]
6.	Джoвaнни et al.	2013137656/15	Effervescent composition in solid form for use in vaginal applications for treating vaginal infections.	*L. plantarum*, *L. pentosus*, *L. casei*, *L. casei* ssp. *paracasei*, *L. rhamnosus*, *Lactobacillus acidophilus*, *L. delbrueckii*, *L. delbrueckii* ssp. *bulgahcus*, *L. delbrueckii* ssp. *delbrueckii*, *L. fermentum*, *L. gasseri*, *L. reuteri*, *Bifidobacterium longum*, *B. bifidum*, *B. breve*, *B. animalis* ssp. *lactis*, *B. adolescentis*, *B. pseudocatenulatum*, *B. eatenulatum* or *B. infantis*	Russian Patent	20 June 2016	Improved stability and a decrease in the capping effect of the tablet while maintaining the distribution of probiotics in the vaginal cavity.	[162]
**Vitamin**								
				**Vitamins Used**				
1.	Aдиcoвнa et al.	2008112493/15	Remedy for treatingBVand method fortreatment.	Ascorbic acid, pantothenic acid, riboflavin, thiamine, vitamin B12	RussianPatent	10 May 2009	Recovery of normal microflora of the vagina and cleaning.	[163]
2.	Qingjun et al.	201910680707.4	Probiotics composition for improving health of urogenital system of women and preparation method of composition.	Probiotics (cranberries, *Hibiscus sabdariffa*, *Lactobacillus*, collagen peptide) and vitamin C	China Patent	10 September 2019	With the help of vitamin C and probiotics, immunity has been increased; the ecological balance of the vagina can be maintained with the help of freeze-dried powder of *Lactobacillus*.	[164]
3.	Weibin et al.	202110362861.4	Female vagina-tightening capsule and preparation process thereof.	Olive, radix *Sophorae flavescentis*, Fructus cnidii, Saffron crocus, radix *Arnebiaeseulithospermi*, radix stemonae, Fructus kochiae, a cortex Phellodendri extract, *Resina draconis* extract, *Stiff silkworm* extract, *Borneol* extract, and vitamin E	China patent	28 May 2021	The intervention had an antibacterial and anti-inflammatory effect, helped in repairing vaginal tissues, and had no toxicity and/or irritation.	[165]
**Protein**								
				**Proteins Used**				
1.	Tingtao et al.	201810873678.9	Construction of *L. crispatus* BT1386 and application in treatment.	CXCL12 (human and mouse chemokine)	China Patent	18 December 2018	Improvement in the BV treatment by restoration of normal vaginal flora helps in tissue repair and decreases inflammation	[166]

BV—Bacterial Vaginosis, EO—Essential Oil, TTO—Tea Tree Oil.

## Data Availability

No new data were created or analyzed in this study. Data sharing is not applicable to this article.

## Supplementary Materials

The following supporting information can be downloaded at: https://www.mdpi.com/article/10.3390/microorganisms13102411/s1, Table S1: PRISMA checklist for systematic review. Source: Page MJ, et al. BMJ 2021;372:n71. doi: 10.1136/bmj.n71. Reference [167] are cited in the supplementary materials.

## Abbreviations

The following abbreviations are used in this manuscript:

BV	Bacterial vaginosis
MDR	Multidrug resistance
MTZ	MTZ
SLD	Sialidase
ECM	Extracellular matrix
MPO	Myeloperoxidase
CFU	Colony-forming unit
AV	Aerobic vaginitis
BVAV	Bacterial vaginosis–aerobic vaginitis
MIC	Minimum inhibitory concentration
FICI	Fractional inhibitory concentration index
LAE	Lauramide arginine ethyl ester
MBC	Minimum bactericidal concentration
MFC	Minimum Fungicidal Concentration
GML	Glycerol monolaurate
LAB	Lactic acid-producing bacteria
HCY	Homocysteine
IZ	Inhibition zone
EO	Essential oil
NPs	Nanoparticles
